# Spen limits intestinal stem cell self-renewal

**DOI:** 10.1371/journal.pgen.1007773

**Published:** 2018-11-19

**Authors:** Maheva Andriatsilavo, Marine Stefanutti, Katarzyna Siudeja, Carolina N. Perdigoto, Benjamin Boumard, Louis Gervais, Alexandre Gillet-Markowska, Lara Al Zouabi, François Schweisguth, Allison J. Bardin

**Affiliations:** 1 Institut Curie, PSL Research University, CNRS UMR 3215, INSERM U934, Stem Cells and Tissue Homeostasis group, Sorbonne Université, UPMC Univ Paris 6, Paris, France; 2 Discngine, Paris, France; 3 Institut Pasteur, Dept of Developmental and Stem Cell Biology, Paris, France; 4 CNRS, UMR3738, Paris, France; University of California, Los Angeles, UNITED STATES

## Abstract

Precise regulation of stem cell self-renewal and differentiation properties is essential for tissue homeostasis. Using the adult *Drosophila* intestine to study molecular mechanisms controlling stem cell properties, we identify the gene *split-ends* (*spen*) in a genetic screen as a novel regulator of intestinal stem cell fate (ISC). *Spen* family genes encode conserved RNA recognition motif-containing proteins that are reported to have roles in RNA splicing and transcriptional regulation. We demonstrate that *spen* acts at multiple points in the ISC lineage with an ISC-intrinsic function in controlling early commitment events of the stem cells and functions in terminally differentiated cells to further limit the proliferation of ISCs. Using two-color cell sorting of stem cells and their daughters, we characterize *spen*-dependent changes in RNA abundance and exon usage and find potential key regulators downstream of *spen*. Our work identifies *spen* as an important regulator of adult stem cells in the *Drosophila* intestine, provides new insight to Spen-family protein functions, and may also shed light on Spen’s mode of action in other developmental contexts.

## Author summary

A fundamental challenge is to identify genes that have essential functions in controlling adult stem cells. Here, we use the *Drosophila* intestinal stem cells as a model of adult stem cells. Through a genetic screen strategy designed to reveal important stem cell regulators in an unbiased manner, we uncovered the gene *split-ends* or *spen* as a key factor required to limit stem cell numbers in the intestine. Spen is part of a conserved family of genes encoding proteins with RNA binding motifs. Our findings suggest that *spen* acts at an early step in stem cell commitment limiting stem cell fate acquisition and further controls stem cell proliferation non-autonomously in terminally differentiated cells. By assessing the effects of *spen* on RNA transcript levels and exon usage, we find that Spen controls a number of genes encoding proteins with similar functions, some of which may explain previously described roles of *spen* during development. Our study provides novel insight into stem cell regulation and function of Spen-family proteins.

## Introduction

During development, pluripotent stem cells will give rise to all of the different cell types present in the organism. Adult stem cells have more limited plasticity and play essential roles in tissue homeostasis and regeneration by both renewing the differentiated cells as well as maintaining the stem cell pool. Defining the mechanisms governing stem cell self-renewal and differentiation is essential for understanding both organism development as well as tissue maintenance and regeneration.

**Fig 1:**
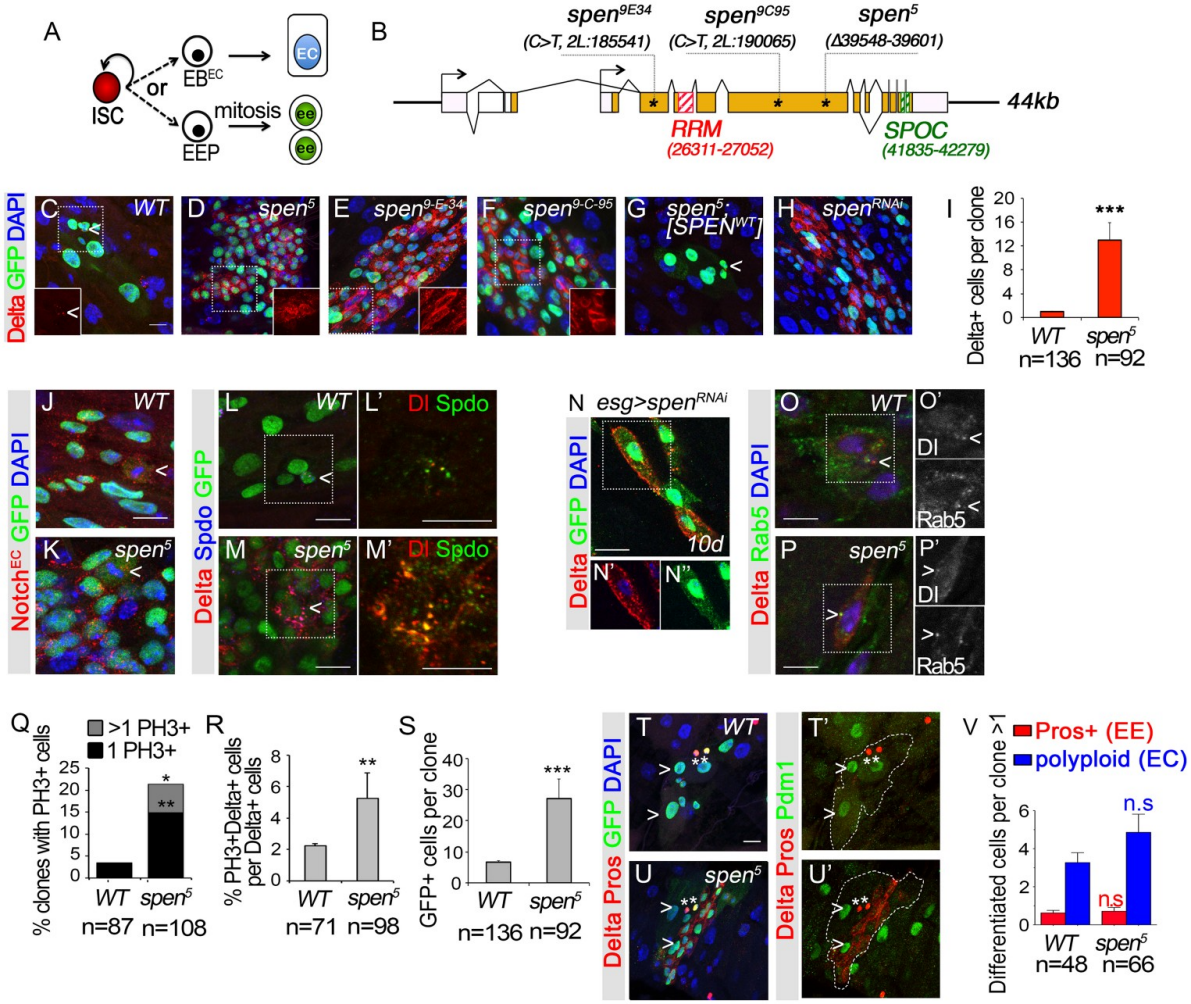
*spen* inactivation leads to an accumulation of intestinal stem cell-like cells in the adult midgut. **(A)** Intestinal stem cells (ISCs) produce post-mitotic progenitor cells, called enteroblasts (EBs) that directly terminally differentiate. Notch activated EBs (EB^EC^) produce enterocytes (ECs) whereas enteroendocrine cells (EEs) are derived from ISCs in a Notch-independent manner via an EE progenitor (EEP) intermediate, which is thought to divide once. **(B)** *spen* gene encodes a protein characterized by 3 RNA recognition motifs (RRM) in the N-terminus (RED), and a SPOC (Spen Paralog Ortholog C-terminus) domain in the C-terminus (GREEN), both included in the different *spen* isoforms. The three mutant alleles *spen*^*9E34*^, *spen*^*9C95*^ and *spen*^*5*^ have premature stop codons, which are frequently targeted by nonsense-mediated decay. The *spen*^*9E34*^ allele has a C>T substitution (2L:185541). The *spen*^*9C95*^ allele has a C>T substitution (2L:190065). The *spen*^*5*^ allele has nucleotide deletion (2L:192716–192769). **(C)** *wild-type* (*WT*), **(D)** *spen*^*5*^, **(E)** *spen*^*9E34*^ and **(F)** *spen*^*9C95*^ mutant clones 5d AHS marked by GFP (GREEN), Dl, (ISCs, RED), DAPI (BLUE). Insets show RED channel only. **(G)** A transgenic BAC construct containing the *spen* wild-type genomic region suppressed *spen*^*5*^ phenotypes in 5 days MARCM clones. **(H)** *spen*^*RNAi*^ (VDRC#KK-108828) in MARCM clones (GFP, GREEN; DAPI, BLUE), 5 days. **(I)** Quantification of Dl+ cells per clone, 5 days AHS. **(J)** wild-type and **(K)** *spen*^*5*^ mutant clones had ISCs (mitotic cells, arrow heads) expressing the Notch receptor (RED in J, K). **(L, L’)** wild-type clones or **(M, M’)** *spen*^*5*^ mutant clones contained ISCs co-labeling with Dl (RED) and Sanpodo (Spdo, GREEN, L’, M’). A single focal plane is shown. An additional 2X zoom is shown in L’, M’. (**N-N”**) *spen*^*7RNAi*^ expression in *esg*+ cells. A single focal plane is shown. (Delta, RED; cytoplasmic GFP, GREEN; DAPI, BLUE), 10 days. **(O, O’)** wild-type clones or **(P, P’)** *spen*^*5*^ mutant clones contained ISCs co-labeling with Dl (RED) and Rab5::YFP. (GREEN, I’, J’**)**. A single focal plane is shown. **(Q)** Quantification of the % of wild-type and *spen*^*5*^ mutant clones with mitotic cells (PH3), 5 days AHS. Fisher’s test. **(R)** Quantification of the number of PH3+ Dl+ cells per Dl+ cells. **(S)** Quantification of clone size in wild-type and *spen*^*5*^ mutant clones. **(T, T’)** Enterocyte (EC) and enteroendocrine (EE, Pros, nuclear RED) cells were produced in wild-type and **(U, U’)** *spen*^*5*^ mutant clones, clones marked by GFP (GREEN); EE (Pros, nuclear red); ISC (Delta, membrane RED); DAPI (BLUE). **(V)** Quantifications of EE and ECs per clone. When not specified, a non-parametric Mann-Whitney test was performed. Error bars represent the Standard Error of the Mean (sem). p<0.05, *. p<0.01, **. p<0.001, ***. p<0.0001, ****. Scale bar:10μm.

The adult *Drosophila* intestine is an attractive model to study adult stem cells as it provides a genetically tractable system with many similarities to other tissues such as the mammalian intestine and lung [1]. The fly intestine is renewed by intestinal stem cells (ISCs), which produce progenitor cells that differentiate into terminally differentiated polyploid absorptive enterocytes (ECs) and diploid secretory enteroendocrine cells (EEs) [2, 3] **(**Fig 1A). A majority of ISC divisions produce EC cells via post-mitotic enteroblast (EB) precursors. Recent findings propose that EEs cells may originate from rare ISC daughter cells that divide once to produce a pair of EE cells [4, 5]. Thus, ISCs are the primary dividing cell type in the intestine. In normal homeostasis, the proliferative status of the ISC is largely controlled through signaling of the EGFR, Jak/Stat, Insulin receptor [6–11] and reviewed in [12]. In addition, in response to epithelial damage, ISCs receive additional proliferative signals promoting tissue renewal [6, 7, 13–23] and see [24] for review). While proliferative control is ensured by the aforementioned signaling pathways, ISC differentiation is largely controlled by Notch signaling [2, 3, 25, 26]. Delta-Notch signaling between stem cell daughters results in Notch activation in EB progenitors that will further promote differentiation into EC cells, whereas inhibition of Notch by Numb and expression of Scute promotes EE fate acquisition [4, 25, 27]. The ISC itself, has low or no Notch signaling, thereby retaining ISC fate. Thus far, outside of canonical Notch signaling components, little is known about the mechanisms controlling ISC fate acquisition leading to robust asymmetric fate acquisition after ISC division.

In an EMS-based genetic screen, we have identified the gene *split-ends* (*spen*) as a regulator of *Drosophila* adult intestinal stem cells, required to limit their production. The SPEN protein family is broadly conserved in plants and *Metazoa* and mediates functions in RNA splicing, polyadenylation, and transcriptional regulation via its RNA binding motifs (RRM) and a SPEN Paralog and Ortholog C-terminal domain (SPOC) protein interaction domain (Fig 1B) [28–30]. There are three mammalian SPEN-family paralogs: SPEN/SHARP, and two family members RBM15/OTT1 and RBM15B/OTT3 encoding smaller proteins. SPEN is known to mediate transcriptional silencing *via* physical association with the nuclear receptor corepressor components including NCoR1, and HDACs [28, 31], while the smaller proteins RBM15 and RBM15B have been primarily found to be associated with splicing regulation [32, 33]. Interestingly, recent studies in mouse embryonic stem cells have demonstrated that RBM15 and RBM15B interact with the RNA m^6^A methyltransferase complex, which facilitates m6A methylation of the non-coding RNA XIST [34, 35]. Consequently, RBM15 and RBM15B are essential for X-inactivation, which also requires SPEN and associated HDAC activities [35–39].

In *Drosophila*, the *Rbm15* paralog Spenito physically associates with components of the RNA m^6^A methylation complex and has been suggested to facilitate m6A RNA methylation and splicing, playing an important role in sex determination [33, 40, 41]. *Drosophila* Spen has diverse roles in development, regulating processes such as cell proliferation, differentiation, cell death, axon guidance, and fat metabolism [42–51]. In mouse hematopoietic stem cells (HSCs), *Rbm15* is essential for return to the quiescent state after stress induction [52]. The functions of SPEN family proteins in other adult stem cell populations have not yet been elucidated. While SPEN family proteins clearly play essential functions during development and in adult tissues, Spen’s molecular targets in both flies and mammals are still poorly understood.

We have identified *spen* in a genetic screen and characterized its function in adult intestinal stem cells. The clonal inactivation of *spen* in stem cell lineages led to increased production of stem cells when compared to control lineages. In addition, *spen* mutant ISCs showed more abundant levels of Delta protein expression and ectopic localization to the cell surface membrane. Targeted knock-down of *spen* in different cell types revealed that it has both stem cell autonomous and non-autonomous roles in controlling ISCs. Despite these defects, *spen* mutant stem cells were still capable of terminal differentiation. We further explored its role in ISCs and EBs by comparing differential gene expression and differential exon usage of wild-type and *spen-*knocked down conditions. We found that *spen* altered transcript abundance and exon usage, suggesting that Spen impacts both transcription and splicing of genes, candidates for downstream regulators of ISCs. Importantly, our work identifies *spen* as a novel regulator acting in multiple cell types in the intestine to control ISC numbers and proliferation.

## Results

### *spen* limits intestinal stem cell number in the adult midgut

In ongoing EMS-induced genetic screens in the lab for defects in ISC proliferation, self-renewal and differentiation, we identified two lines (9E34 and 9C95) which caused altered numbers of diploid cells. They failed to complement each other and mapped to the same genomic region by deficiency mapping. Genes in the region were tested and both lines failed to complement previously identified alleles of gene *split-ends* (*spen)*: *spen*^*3*^ and *spen*^*5*^ [43]. *spen* encodes 8 isoforms of similar sequence of approximately 5500 amino acids (aa). 3 RRMs (RNA Recognition Motifs) domains are found N-terminally and a SPOC domain C-terminally (Spen paralog and ortholog C-terminal). *spen*^*5*^ was previously characterized as deleting nucleotides 39548–39601 (2L:192716–2L:192769) resulting in a frame shift and early termination [43]. Sequencing of the mutant lines *spen*^*9E34*^ and *spen*^*9C95*^ identified nonsense mutations, affecting nucleotides 2L:185,541 and 2L:190,065 respectively (Fig 1B). RNAs with non-sense mutations are frequently degraded via non-sense mediated decay, however, if translated, these RNAs would result in truncated proteins. *spen*^*9E34*^ allele would encode a putative protein of 630 aa, including the RRM domains. *spen*^*9C95*^ and *spen*^*5*^ would potentially encode putative truncated proteins of approximately half the wild-type protein size, of respectively 1961 aa, and 2827 aa (Fig 1B). These alleles all likely function as loss of function alleles as 1) they all result in similar phenotypes, 2) these mutant phenotypes are like those of expressing *spen* RNAi (see below), and 3) *spen*^*5*^ mutant phenotypes can be rescued by a single copy of a genomic rescue construct, arguing against dominant neomorphic function of a truncated mutant protein (see below). Published RNAseq data suggest that Spen is likely expressed at low levels in all epithelial cell types of the gut [53] (http://flygutseq.buchonlab.com/). Thus, despite apparent low levels of expression, *spen* was identified in our genetic screen as being critical to control diploid cell number in the adult intestine.

To further investigate how *spen* affects the ISC lineage in the *Drosophila* posterior midgut, we used previously described markers of ISCs to assess the effect of *spen* inactivation on cell fate using the Mosaic Analysis with Repressible Cell Marker (MARCM [54]) technique in adult ISCs. In this method, a heat shock is given in adult animals to induce expression of a Flipase, which then promotes exchange of homologous chromosomes containing FRT sequences, thereby leading to the production of either wild-type or mutant ISC lineages marked through GFP expression. In the adult intestine, marked lineages are induced preferentially in ISCs, as the ISC is the primary dividing cell type in the adult intestine. In order to characterize the *spen* phenotype, we first examined the expression of the stem cell marker Delta (Dl) in *spen* mutant and knockdown stem cell clones (Fig 1C–1I). 5 days after heat shock (AHS), wild-type stem cell clones contained 1.0 Dl+ ISC on average per clone (Fig 1C and 1I). In contrast, *spen*^*5*^ mutant stem cell clones contained 13.0 Dl+ cells per clone (Fig 1D and 1I). Similar phenotypes for *spen*^*9E34*^ and *spen*^*9C95*^ were observed (Fig 1E and 1F). In addition, the aberrant increase of Dl+ cells was rescued upon insertion of a *pACMAN BAC* construct containing the *spen* wild-type genomic region, and was phenocopied when clones expressed *spen*^RNAi^ (Fig 1G and 1H). The Dl+ cells produced in *spen* mutant clones also expressed additional markers of ISCs: the Notch receptor (Fig 1J–1K), Sanpodo (Spdo; Fig 1L–1M’) [26], which marks both ISCs and the EB daughter cells [3]. Thus, *spen* mutants accumulate Dl+ cells that share additional features with wild-type stem cells. In addition, the level of Dl protein at the cell membrane and in endocytic vesicles was markedly higher in *spen* mutants than in wild-type (Fig 1N and 1O–1P’).

Consistent with an increased number of stem cells upon *spen* inactivation, mutant clones contained more Phospho Histone H3+ (PH3+) cells than wild-type: whereas only 3.4% of wild-type clones contained one PH3+ cell, 21.3% of *spen* mutant clones had at least one (Fig 1Q). This is also consistent with the significant increase in the proportion of dividing stem cells in *spen* mutants: in the wild-type population 2.2% of Dl+ were PH3+ whereas 5.2% of Dl+ were PH3+ in the *spen* population (Fig 1R). In agreement with an alteration in proliferation, *spen* inactivation led to larger clones containing 27.2 cells per clones compared to wild-type clones, which contained 6.7 GFP+ cells in total (Fig 1S). Altogether, our data indicated that inactivation of *spen* results in larger clones and that had an excess number of ISCs, suggesting that Spen is required to limit the number of stem cells.

In contrast to ISC numbers, cell fate specification of terminally differentiated cells was not affected in *spen* mutant clones: at 5 days AHS, wild-type clones contained 3.3 ECs (marked by nuclear size >7μm and Pdm1 expression) and 0.6 EEs (marked by Prospero expression) per clone on average (Fig 1T and 1T’). Similarly, *spen* mutant clones contained both types of terminally differentiated cells with 4.9 ECs and 0.7 EEs per clone (Fig 1U and 1V). Therefore, we conclude that terminally differentiated cells are produced in absence of *spen* function.

### *spen* acts in stem cell autonomously and non-autonomously to regulate stem cells

**Fig 2:**
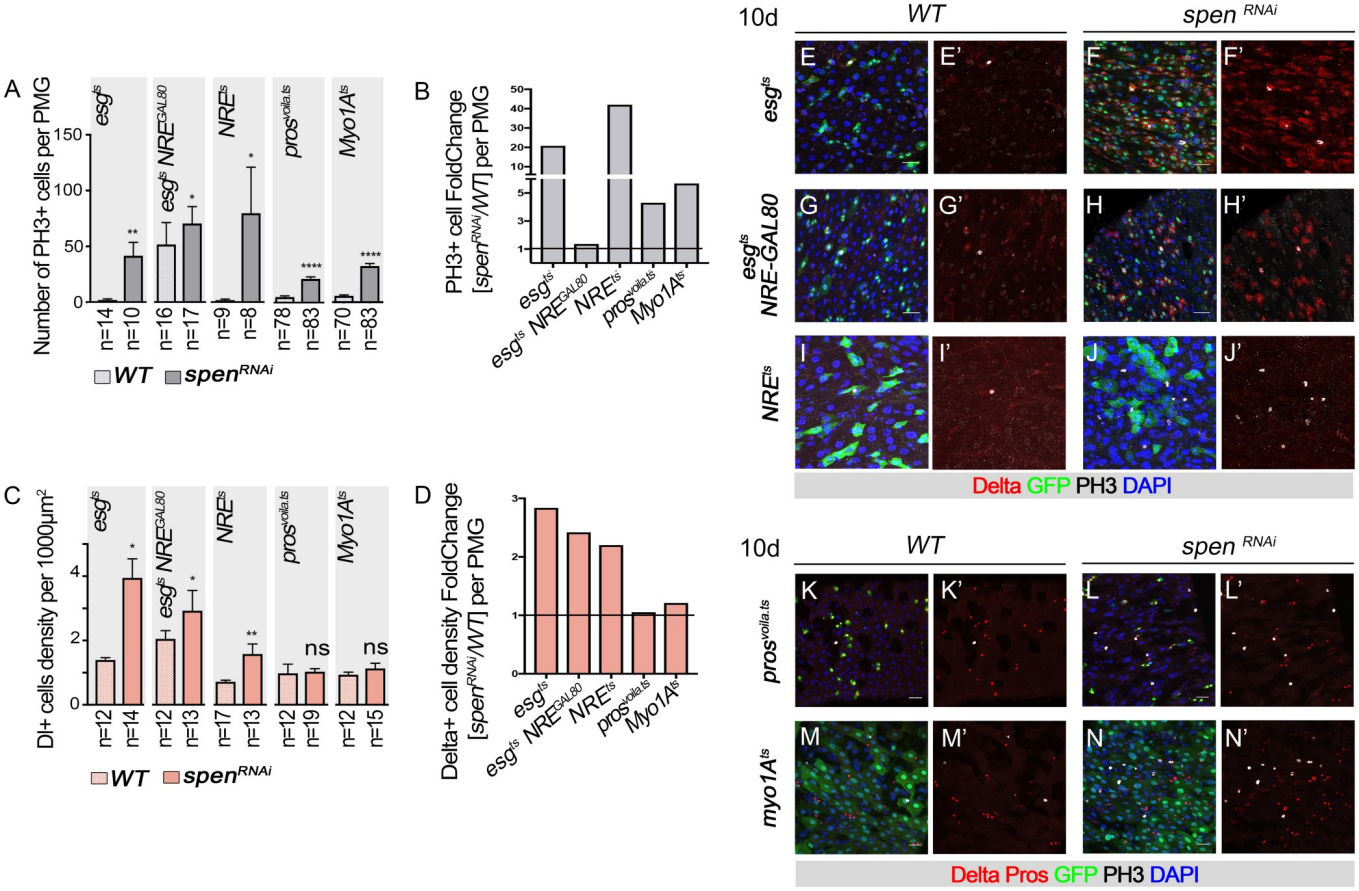
Spen has stem cell autonomous and non-autonomous functions to control ISC numbers and proliferation state. (**A-D)** Quantification of E-N’ (**A**) the number of mitotic cell (PH3+ cells) or (**B**) relative increase in proportion per posterior midgut (PMG) in the indicated genotypes. (C) the density of Dl+ ISCs or **(D)** relative increase. **(E, E’, G, G’, I, I’, K, K’, M, M’)** wild-type UAS-GFP control and **(F,F’, H, H’, J, J’,L, L’, N, N’)** *UAS-spen*^*RNAi*^ expressed for 10 days at 29°C. An increased number of PH3+ cells and Dl+ ISCs was detected upon expression of *UAS-spen*^*RNAi*^ in ISCs and EBs **(E- F’)**, ISCs only **(G-H’)**, and EBs only (**I- J’**; using esg^ts^, *esg*^*ts*^ *combined with NRE-GAL80 –*to block expression in Notch active EB cells, or *NRE*^*ts*^, respectively). (**K-N’**) An increase in PH3+ cells was also found upon expression of *UAS-spen*^*RNAi*^ in Enteroendocrine cells (*pros*^*voila*.*ts*^) or in Enterocytes using *myo1A*^*ts*^. GFP in GREEN marks cell type expression, Delta+ ISCs, RED, mitotic cells (PH3+, Gray), DNA (DAPI, BLUE). Scale bar: 20μm.

*spen* is expressed in all epithelial cell types of the gut [53]. To investigate in which cell type *spen* function is required to control intestinal stem cell production, we expressed *spen*^*RNAi*^ in a cell-*type* specific manner using the TARGET system [55]. The downregulation of *spen* simultaneously in both ISCs and EBs using the *esg-GAL4*, *tub-GAL80*^*ts*^ line (*esg*^*ts*^) led to an enrichment of Delta protein at the membrane in ISCs as well as an increased density and proliferation of ISCs (PH3+ cells; Fig 2A–2F’). Quantification revealed a 2.8X increase in the number of ISCs per 1000μm^2^ and a 20X increase in the number of PH3+ cells on average per posterior midgut upon *spen* depletion in ISCs and EBs (Fig 2A–2D).

The knockdown of *spen* in ISCs-only using *esg-GAL4*, *tub-GAL80*^*ts*^, *NRE-GAL80* line, similarly led to altered Dl membrane location in ISCs, and increased numbers (2.4X) and proliferation of ISCs (1.3X), measured by the density of Dl+ cells and number of PH3+ cells (Fig 2A–2D and 2G–2H’). Because the effect on ISC proliferation was less pronounced when *spen* was knocked down in ISCs only as compared to ISCs and EBs, we hypothesized that *spen* may also function in EBs to limit ISC proliferation. To further explore a function of *spen* in EBs that could limit non-cell autonomously ISC proliferation, we knocked down *spen* function in EBs using *NRE-GAL4*. Consistent with this notion, EB-knockdown of *spen* led to a 2.2X increased number of Dl+ and a 42X increase in the number of PH3+ cells per posterior midgut (Fig 2A–2D and 2I–2J’). Importantly, there was no impact on Dl membrane localization in ISCs (Fig 2I–2J’), indicating that 1) this activity of *spen* is ISC autonomous and 2) that *spen* has additional effects on limiting ISC proliferation and ISC numbers that are independent of its activity in the ISC.

We then further explored potential non-cell autonomous functions of *spen* by examining the effects of the knockdown of *spen* in terminally differentiated EE and EC cells. Expression of *spen*^*RNAi*^ in enteroendocrine cells using the *pros*^*voila*^*-GAL4*, *tub-GAL80*^*ts*^ line, or in EC cells using the *Myo1A-Gal4*, *tub-Gal80*^*ts*^ did not have a major impact on the density of Dl+ ISCs or Delta membrane localization in ISCs (Fig 2A–2D and 2K–2N’). Interestingly, we found that while ISC numbers were not altered, the knockdown of *spen* in ECs and EEs did lead to increased ISC proliferation suggesting a non-autonomous function of *spen* in controlling ISC proliferation from EEs and EC cells (Fig 2A–2D and 2K–2N’). Indeed, *spen* depletion in EEs led to a 3X increase, whereas inactivation in ECs led to a 4X increase in PH3+ cells. (Fig 2B).

We did notice, however, that both of these drivers, showed some leakiness of expression in Dl+ ISC cells (S1 Fig). Consistent with this, we observed that upon *spen* knockdown with *pros*^*voila*^*-GAL4* and *Myo1A-*Gal4, as well as NRE-Gal4 line, rare Dl+ ISCs had strong Dl signal at the membrane and showed GFP expression indicating driver leakiness (S1 Fig). However, we believe that the limited number of ISC cells with leaky expression cannot explain the dramatic effect on ISC proliferation noted above. Thus, *spen* activity in ECs and EEs limits ISC proliferation.

Therefore, through this series of experiments, we conclude that *spen* has multiple functions in controlling ISCs: an ISC-intrinsic role in controlling Dl protein accumulation at the membrane, ISC fate and ISC proliferation; a function in the EB to non-autonomously regulate ISC fate and proliferation; and roles in terminally differentiated cells that limit ISC proliferation.

### *spen* acts upstream or parallel to Notch activation in control of ISC numbers

Our data thus far indicated that *spen* activity has different functions: controlling Dl accumulation on the cell membrane (in ISCs), limiting ISC number (in ISCs and EBs), and restraining proliferation (in ISCs, EBs, EEs and ECs). Importantly, these separate activities can occur independently of each other, suggesting that they involve distinct mechanisms.

**Fig 3:**
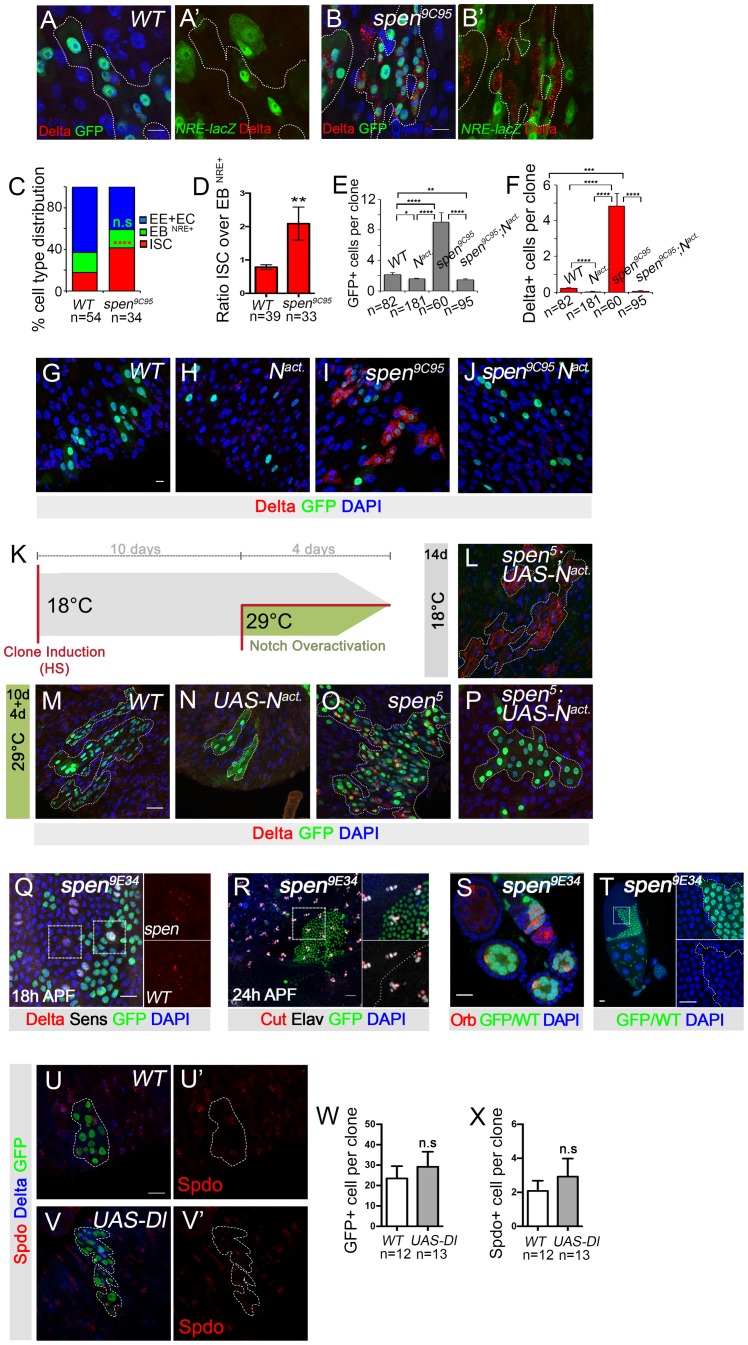
*spen* controls an early stem cell commitment event. **(A, A’)** Wild-type and **(B, B’)** *spen*^*9C95*^ 5 day AHS MARCM clones showed activation of the Notch pathway, as detected by NRE-lacZ (Notch Responsive Element, also called *Su(H)-GBE-LacZ*) [57], ßGal, Green) (GFP, GREEN; DAPI, BLUE). **(C)** Quantification of cell type distribution, ISCs (Dl+ cells) in RED; EBs (EC precursors, NRE-LacZ+, in GREEN), and combined other cells representing mostly EEs and ECs, in BLUE. While the production of EB (NRE+) cells per clone was not affected upon *spen* inactivation, the proportion of Dl+ ISC-like cells significantly increased *spen*^*9C95*^ clones. **(D)** Upon *spen* inactivation, ISC-like cells were more produced compare to EB (NRE+) cells. **(E)** Quantification of the number of GFP+ cells per clone or **(F)** Dl+ cells per clone for G-J (below). Single cell clones have been included in these quantifications (E-F). **(G)** Control wild-type clones produce multi-cell clones, whereas **(H)** the expression of an activated form of the Notch receptor (*UAS-N*^*act*^) promoted differentiation. **(I)** Similarly, *spen* mutant ISCs produced multi-cell clones whereas **(J)** the expression of *UAS-N*^*act*^ drove differentiation and resulted in single-cell clones. (Dl+, RED; clones marked by GFP in GREEN; DAPI in BLUE (G-J). **(K)** Scheme of experimental set-up: Mitotic clones were first generated, then *UAS-N*^*Act*^ was expressed after 10 days in these clones by releasing Gal80ts inhibition at 29°C during 4 days. **(L)** *spen*^*5*^*; UAS-N*^*Act*^ genotype at 18°C where Gal80ts was active therefore *UAS-N*^*Act*^ was not induced. **(M-P)** At 29°C, Gal80ts became inactive in **(M)** wild-type clones, **(N)** those expressing *UAS-N*^*Act*^, **(O)** *spen*^*5*^ clones, and **(P)** *spen*^*5*^*; UAS-N*^*Act*^. Delta+ in RED; clones GFP in GREEN; DAPI in BLUE. **(Q)** Neither *spen*^*5*^ nor **(R)** *spen*^*9C94*^ mutant clones showed defects in sensory organ specification, lineage decisions, or Dl protein accumulation at 18h after pupation (Q) or 24h after pupal formation (APF) (R). **(S)** Germline and (**T**) follicular cell clones of *spen*^*9C94*^ (lack of GFP, GREEN) did not present visible phenotypes akin to defects in Notch signaling. (**U, U’**) Control or (V, V’) UAS-Delta overexpressing clones. Spdo+ in RED; Delta in BLUE; clone GFP in GREEN. (W) Quantification of number of cells per clone U-V’. (X) Quantification of number of Spdo+ cells per clone. For all quantifications a non-parametric Mann-Whitney Two-Way ANOVA test was performed. Error bars represent the Standard Error of the Mean (sem). p<0.01, **. p<0.001, ***. p<0.0001, ****. Scale bars: 10μm, except in T where is 20μm.

In order to better understand the role that *spen* plays in controlling ISC fate and limiting ISC numbers, we then focused on a potential interaction with the Notch pathway, the major pathway implicated in controlling cell fate decisions in the ISC lineage. We hypothesized that *spen* may function through Notch regulation to mediate cell fate decisions and asymmetric fate outcomes. The inactivation of the Notch pathway causes an accumulation of ISC cells and excess numbers of EE cells [2, 3]. Nevertheless, our previous work has shown that lowering, but not completely inactivating Notch signaling can increase the number of ISCs without impacting EE and EC cell numbers [26], similar to the phenotype observed here for *spen* mutant clones. In addition, links between the Spen family proteins and Notch signaling have been demonstrated in other tissues [31, 49, 50, 56]. Thus, *spen* could promote asymmetric outcomes of the cell fate decisions following stem cell divisions by influencing Notch pathway signaling. We therefore explored the possibility that deregulated Notch signaling occurred upon *spen* inactivation by assessing a reporter of Notch transcriptional activity (*NRE-LacZ*, NRE+ cells) in *spen* mutants. The NRE reporter is a synthetic construct with three copies of the SPS site (for Su(H) Paired Sites, binding sites for the Notch activity-dependent transcription factor, Su(H)) that is taken from the *E(spl)m8* regulatory region and combined with three copies of the Grh binding element (Gbe); it likely reports Notch signaling above a given threshold [57]. While in *spen* mutant clones there was an overabundance of Dl+ ISCs that did not have activated Notch, consistent with an increase in stem cells, many cells in these clones still activated the Notch pathway (Fig 3A–3D), further consistent with our results above that terminal differentiation was unaffected. However, these data do not rule out *spen* acting to enhance Notch signaling but not being required for it, affecting a subset of cells receiving Notch signaling. Thus, while *spen* mutant stem cells overproliferated and did not produce as many Notch active EB progenitors per stem cell as wild-type stem cells, they could still derive enough progenitors to give rise to normal numbers of EC cells. The lack of defects detected in EE cell production also imply that *spen* does not alter the production of EE cell precursors derived from the ISC.

Upon cell division, both ISC daughter cells inherit Dl protein present in the ISC mother cell. In a majority of ISC divisions, one of these daughter cells will undergo commitment through activation of Notch signaling and extinguish its expression of Dl and become an EB cell committed towards EC fate [58]. It is possible that ISCs accumulate in *spen* mutants because of a delay in the commitment process, which would result in a decreased number of EBs relative to ISCs. We assessed this by comparing the number of ISCs (Dl+) to the number of EB cells (NRE+). In wild-type lineages, there were 0.79 Dl+ISCs per NRE+ EB cells 5 days AHS. In contrast, upon *spen* inactivation, there were 2.1 ISCs per EB (Fig 3D), suggesting that *spen* mutant ISCs have more symmetric ISC divisions that wild-type ISCs. Thus, while most wild-type stem cell divisions result in asymmetric daughter cell fates [59, 60], in *spen* mutants stem cell divisions, ISC division more frequently leads to symmetric stem cell fate acquisition.

As Spen family members have been implicated in transcriptional regulation, it is possible that *spen* acts to promote reliable activation of Notch target genes either directly or indirectly, as has been suggested in the hematopoietic system [50]. We therefore tested whether *spen* mutant cells could differentiate in response to cleaved active Notch. If *spen* were required for the activation of Notch target genes, the expression of activated Notch would have no effect in *spen* mutants. As previously demonstrated [3], when a cleaved active form of Notch was expressed in wild-type stem cells, it caused their differentiation into ECs generating 1 or 2 cell clones (Fig 3E–3H). *spen* mutant clones contained 4.8 Delta+ cells per clone, while the expression of an active Notch receptor (*UAS-N*^*Act*^) suppressed this to 0.05 Delta+ cells per clone in *spen* and led to smaller clones composed largely of ECs (Fig 3E–3J). To exclude the possibility that overexpression of activated Notch simply forced differentiation of the ISC prior to establishment of the *spen* loss of function phenotype due to residual Spen protein, we repeated the experiment with altered kinetics: *spen* was first inactivated in MARCM clones, then *UAS-N*^*Act*^ was expressed in these clones by releasing Gal80ts inhibition at 29°C (Fig 3K–3P). 14d after *spen* mutant clone induction, indeed stem cells with Delta enrichment at the membrane accumulated (Fig 3L). Note that in this experiment no GFP was detected due to the activity of Gal80ts at 18°C. However, large clones composed of ECs were produced when Notch pathway overactivation was induced for 4d, 10d after *spen* clones had been induced (Fig 3P). Thus, the extra ISC-like cells resulting from *spen* inactivation were forced to differentiate into ECs, when the active Notch form was expressed (Fig 3L–3P). Therefore, *spen* mutant ISCs can respond to Notch pathway activation and differentiate, indicating that *spen* is not essential for Notch target gene activation. This is consistent with our findings above that some *spen* mutant cells can activate Notch signaling.

These data suggest that *spen* may be upstream of, or parallel to, cleavage and activation of the Notch receptor. One important step upstream of Notch activation is the trafficking and endocytosis of its ligand Delta, raising the possibility that the increased numbers of stem cells may result from cell fate defects due to alteration of Delta trafficking or endocytosis. Interestingly, we noticed that Dl protein, but not Sanpodo protein, was more abundant and more strongly localized to the plasma membrane in *spen* mutant stem cells than in wild-type stem cells (Fig 1D and 1L–1N). To test whether this effect on Delta localization was specific to the intestine, we generated *spen* mutant clones in the sensory organ cells of the peripheral nervous system (PNS). We did not find obvious effects on Delta protein levels nor subcellular localization, or lineage specification defects in the PNS (Fig 3Q–3R), or in the ovary (Fig 3S and 3T). Spen’s function on Dl levels is therefore tissue specific.

The membrane accumulation of Delta is known to inhibit, in *cis*, the activity of the receptor Notch present in the same cell [61, 62]. We thus tested whether simply elevating the levels of Dl by overexpression was sufficient to promote accumulation of stem cells using the marker Spdo to assess the number of ISCs. Consistent with observations of others [8, 13, 63], overexpression of Dl in clones led to an enrichment of Delta protein at the membrane, but did not drive accumulation of ISCs or affect stem cell proliferation (Fig 3U–3X). This suggests that the *spen* phenotype is not due to a simple increase in Dl levels, though it could result from altered Dl trafficking dynamics.

All together, these data suggest *spen* has a tissue-specific effect on Dl protein levels and/or trafficking to the cell membrane, and that *spen* limits symmetric cell fate acquisition upon ISC division. This activity impinges upstream or in parallel to cleavage and activation of the Notch receptor.

### *spen* mutant ISCs are less sensitive to lowered EGFR signaling, but are dependent on Akt/Insulin signaling

**Fig 4:**
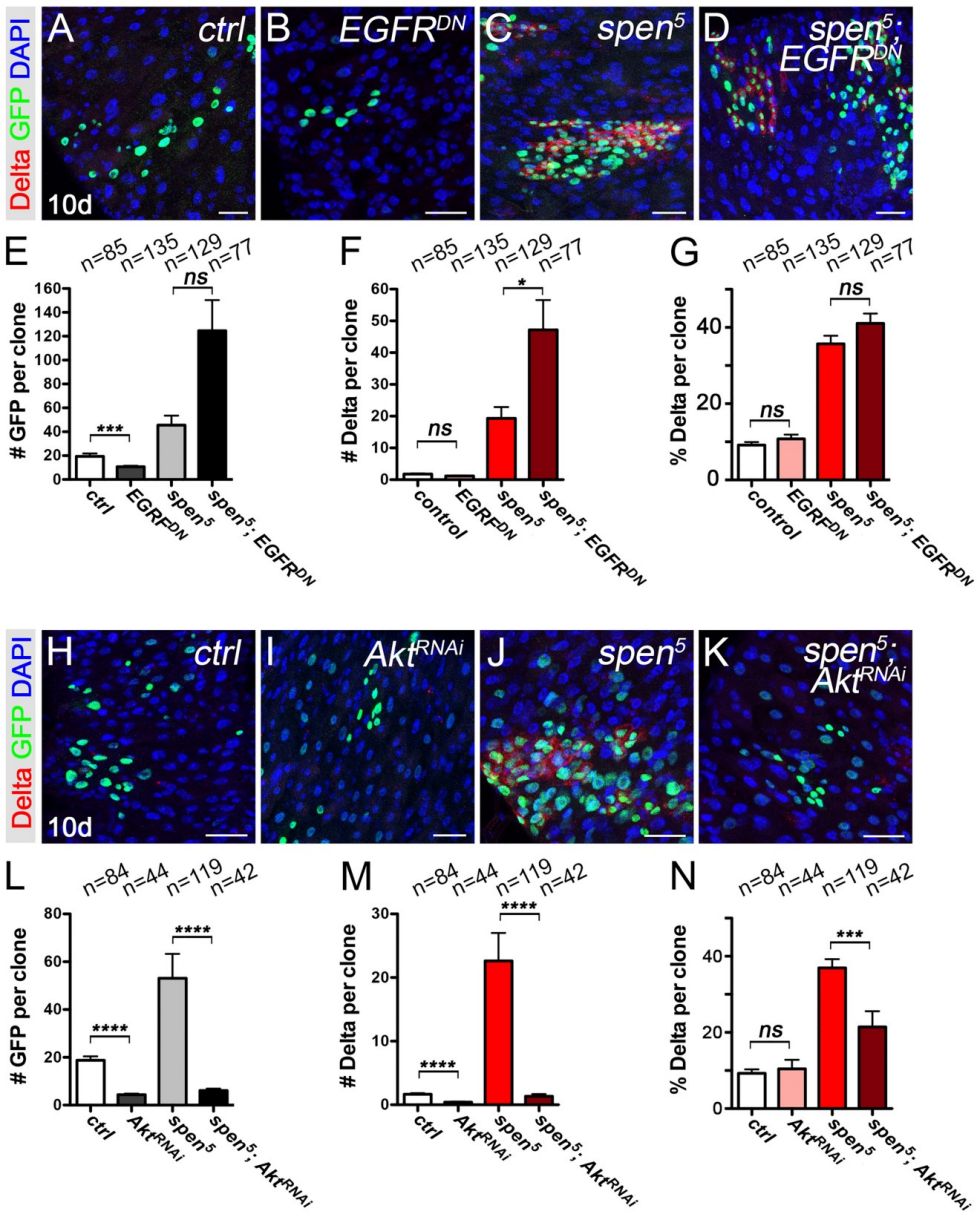
Spen mutants are less sensitive to lowered EGFR signaling, but are suppressed by lowering Akt/Insulin signaling. **(A-D)** Genetic interaction between *spen* and *EGF receptor (EGFR)*. **(A)** Wild-type clones, **(B)**, *EGFR*^*DN*^ clones, **(C)**, *spen*^*5*^ clones, and **(D)** *spen*^*5*^;*EGFR*^*DN*^ clones, 10d after heat shock (AHS). (Delta+, RED; GFP, GREEN; DAPI, BLUE,) **(E)** Quantification of: cells per clone, **(F)** Dl+ cells per clone, and **(G)** Dl cell proportion per clone in A-D. **(H-K)** Genetic interaction between *spen* and *AKT* **(H)** Wild-type clones, **(I)**, *AKT*^*RNAi*^ clones, **(J)**, *spen*^*5*^ clones, and **(K)** *spen*^*5*^;*AKT*^*RNAi*^ clones, 10d after heat shock (AHS). (Delta+, RED; GFP, GREEN; DAPI, BLUE,) **(L)** Quantification of: cells per clone, **(M)** Dl+ cells per clone, and **(N)** Dl cell proportion per clone in A-D. p<0.01, **. p<0.001, ***. p<0.0001, ****. Mann-Whitney Two-Way ANOVA test. One cell clones were excluded in this quantification as only stem cell clones were analyzed. Error bars represent the Standard Error of the Mean (sem). Scale bar: 25μm.

To gain insight into how *spen* regulates ISC proliferation, we tested whether proliferation of *spen*^*5*^ mutant ISCs was dependent on EGFR signaling, a major pathway controlling ISC cell division. As previously reported [9, 64], we found that the expression of a dominant-negative version of EGFR (EGFR-DN) reduced the size of wild-type clones (19.4 cells per clone in controls to 10.7 cells per clone on average in EGFR expressing clones). Interestingly, expression of EGFR-DN did not decrease the size of *spen* mutant clones (Fig 4A–4E). Furthermore, in *spen* mutants expressing EGFR-DN neither the number, percentage of Dl+ ISCs per clone, nor the strong accumulation of Dl at the membrane were suppressed compared to *spen* mutants (Fig 4A–4D, 4F and 4G). Thus, we conclude that *spen* mutant clones are less sensitive than wild-type clones to reduction in EGFR activity. However, as the EGFR-DN likely does not totally inactivate EGFR signaling, further studies combining complete inactivation of EGFR with *spen* will be required to determine whether *spen* mutant stem cells can proliferate in its absence.

In mammalian cells, Spen was recently suggested to influence the activity status of Akt [65], a downstream component of the Insulin pathway known to regulate ISC proliferation [6, 59, 66, 67]. We therefore wanted to test whether inactivating Akt or the Insulin receptor could suppress *spen*-associated phenotypes. We found that reducing the activity of *Akt* decreased both the size of *spen* mutant clones and the accumulation of Dl+ ISCs per clone (Fig 4H–4N). Interestingly, the strong accumulation of Dl at the membrane was not observed in small *spen*^*5*^*; Akt RNAi* cells (Fig 4J and 4K) suggesting that *Akt* is required for the accumulation of Dl in *spen* mutants. Very few clones were recovered of *spen*^*5*^ mutant expressing a dominant negative Insulin receptor, suggesting clone loss. However, a majority of those that were recovered were very small (6.9 *spen*^*5*^, *InR-DN* cells on average compared to 70 cells in *spen*^*5*^ mutant clones; S2 Fig), consistent with the *spen*^*5*^, *Akt RNAi* phenotype described above.

Altogether, these data suggest that the proliferation of *spen* mutant stem cells is less sensitive to levels of EGFR signaling but dependent on Akt and Insulin receptor signaling.

### *spen* controls transcript abundance and differential exon usage

**Fig 5:**
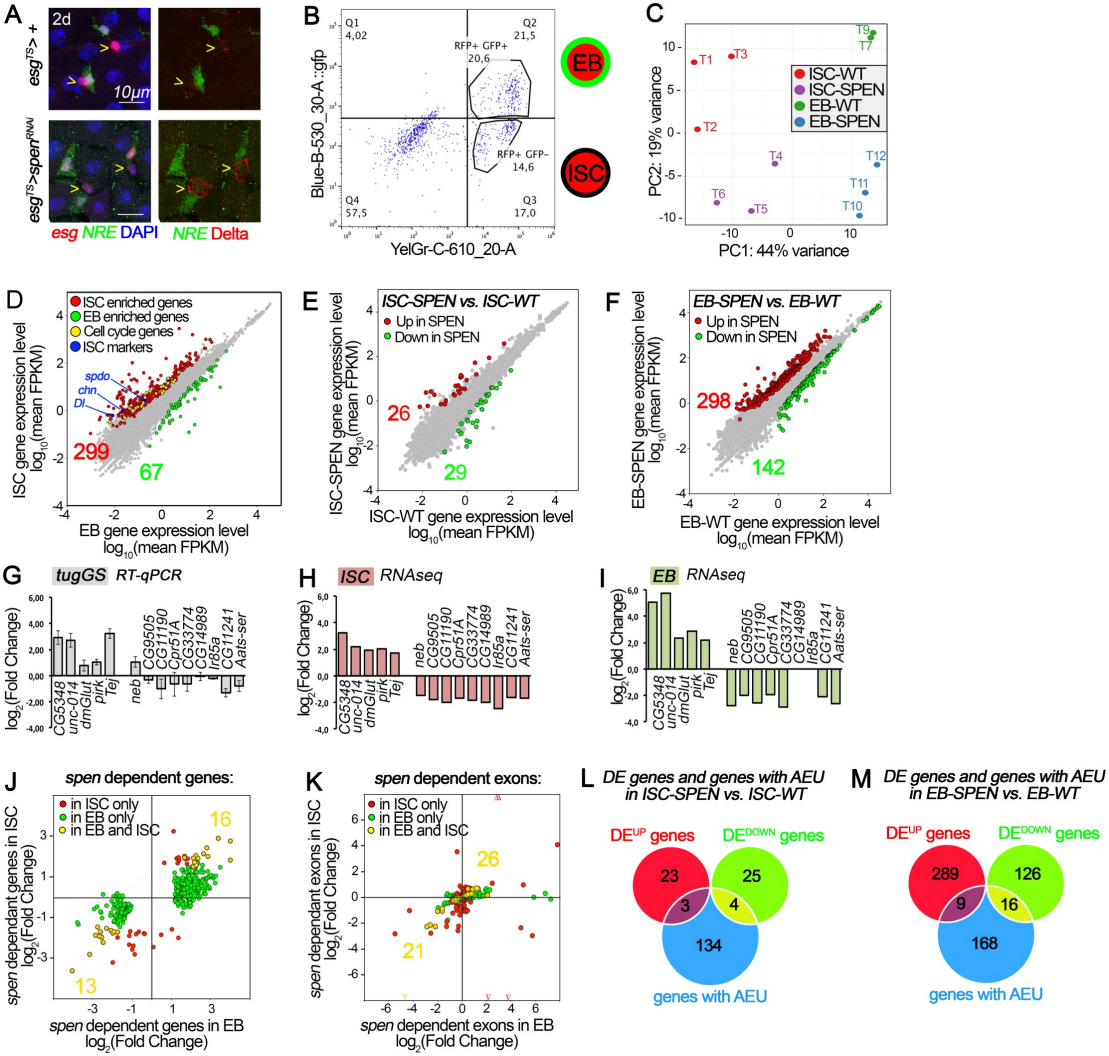
*spen*-dependent transcript identification by RNAseq analysis from two-color FACS sorted *ISC and EB*. **(A)** ISCs and EBs were sorted using a two-color FACS sorting approach following 2 day expression using *esgGAL4*, *Gal80ts* to drive *UAS-RFP* expression in both cells, and *NRE-Venus* (GREEN) to mark only EB cells (EC precursors). Wild-type controls (top panels) showed vesicular Dl staining in esg+ NRE- cells, *spen*^*RNAi*^ (bottom panels) showed membrane accumulation of Dl in esg+ NRE- cells. Scale bar: 10μm. **(B)** Flow cytometry analysis of ISC and EB sorting. The scatterplot revealed two distinct populations: RFP positive (ISCs) and RFP and GFP positive EBs cells. **(C)** Principal component analysis (PCA) based on the 300 top genes with the highest variance clustered our samples in to four distinct groups. **(D)** Differential gene expression comparison between *WT* ISCs and *WT* EBs revealed 366 differentially expressed genes, FDR of 0.05. Genes significantly enriched in ISC (RED), genes significantly enriched in EB (GREEN), known ISC markers (BLUE), cell-cycle related-genes (YELLOW). **(E)** Differential gene expression comparison between *WT vs*. *spen*^*RNAi*^ expressing ISCs or **(F)** EBs; FDR of 0.05. (**G-I**) RT-qPCR validation of *spen-*dependent genes from whole midguts that expressed ubiquitinously *spen*^*RNAi*^ during 2days *(tubGS*), differentially expressed in RNAseq data from (H) ISCs and (I) in EBs. **(J)** Comparison of *spen*-dependent differentially expressed (DE) genes between ISCs and EBs. (**K**) *spen*-dependent differentially used exons. Arrowheads represent exons that are out of the axis range. **(L, M)** Overlap between differentially expressed (DE) genes and genes with alternative exon usage (AEU) in absence of *spen* in ISCs, or **(L)** in EBs (**M**).

We then wanted to better characterize downstream effectors of Spen in ISCs and EBs that may contribute to adult stem cell regulation. Spen family members have been shown to regulate gene expression at transcriptional and post-transcriptional levels [30, 33, 34, 36, 68–70]. We thus assessed the impact of *spen* loss of function on gene expression at transcriptomic and exonic level in ISCs and compared this with EBs. We developed a two-color FACS sorting approach to simultaneously sort ISCs and EBs (NRE+ cells). The *esgGal4*, *Gal80ts* system to express *spen*^*RNAi*^ in both ISCs and EBs, along with RFP to mark these cell populations was used. The EBs were further discriminated using a *NRE-Venus* reporter [71]. Thus, ISCs were RED only and Notch active EBs were RED and GREEN (Fig 5A). Wild-type and *spen*^*RNAi*^ ISCs and EBs were sorted by FACS (Fig 5B). polyA+ RNAs were isolated 2 days after *spen*^*RNAi*^ expression and sequenced. RNAseq analysis was then performed to assess the effect of *spen* inactivation on differential gene expression and exon usage.

In order to validate our RNAseq protocol, we first assessed the similarities between our samples. Principal component analysis (PCA) revealed that our samples clustered in four distinct groups according to their genotype and cell type: WT-ISC, SPEN-ISC, WT-EB and SPEN-EB (Fig 5C). Secondly, we analyzed differential gene expression between sorted wild-type stem cells and wild-type EB cells using DEseq2 [72]. Among the 366 differentially expressed genes identified with a False Discovery Rate (FDR) of 0.05, 299 genes were upregulated in ISCs compared to EBs, including known ISC markers and cell cycle related-genes: *polo* [73], *Cdc2* [73], *Delta* [2, 3], *sanpodo* [26], *charlatan* [74] (Fig 5D, S1 and S2 Tables). In addition, Gene Ontology terminology (GO term) enrichment analysis of the ISC-enriched genes revealed an over-representation of functions and processes associated with cell division regulation and cytoskeleton regulation, reflecting stem cell properties (S3 Table). Interestingly, other over-represented functions were also found associated with regulation of protein localization to nucleus, enzyme activity, signal transduction, and negative regulation of cell differentiation, among others (S3 Table). No specific GO term enrichment could be found with EB-enriched genes. Thus, ISCs and EBs cells were separated by our two-color FACS approach, and this allowed us to reliably identify differentially expressed genes.

SPEN-family proteins play prominent roles in transcription as well as post-transcriptional processes. Therefore, we analyzed the impact of *spen* inactivation on differential gene expression (DEseq2) [72] and differential exon usage (DEXseq) [75] in ISCs and EBs. In ISCs, we found 55 genes were differentially expressed with a FDR of 0.05, of which 26 were up and 29 were down upon *spen* inactivation (Fig 5E, S4 Table). In EBs, 440 genes had significantly altered expression with 298 up and 142 down in *spen*^*RNAi*^ (Fig 5F, S4 and S2 Tables). We validated a number of these genes using RT-qPCR analysis. Indeed, genes that were found up-regulated or down-regulated in ISC and/or EB in our RNAseq data, showed similar relative expression pattern when *spen* was knockdown in whole midgut (compare Fig 5G to 5H and 5I). Interestingly, 29 (13 DOWN and 16 UP) *spen-*dependent genes were similarly de-regulated in the two cell types (Fig 5J and S2 Table). Of note, the observed upregulation of *spen* in ISCs and EBs expressing *spen*^*RNAi*^ was due to the overexpression of the dsRNA (742bp) that targets the exon E14 of *spen* in this experimental set up. Indeed, sequencing reads accumulated in both *sense* and *anti-sense* orientations in this region when *spen*^*RNAi*^ is expressed, but not in WT. Importantly, we did not detect alteration of *Dl* mRNA levels or exon usage, suggesting that the increase in Dl protein detected in *spen* mutant clones is likely due to regulation of Dl protein itself, perhaps during its endocytic trafficking steps.

**Fig 6:**
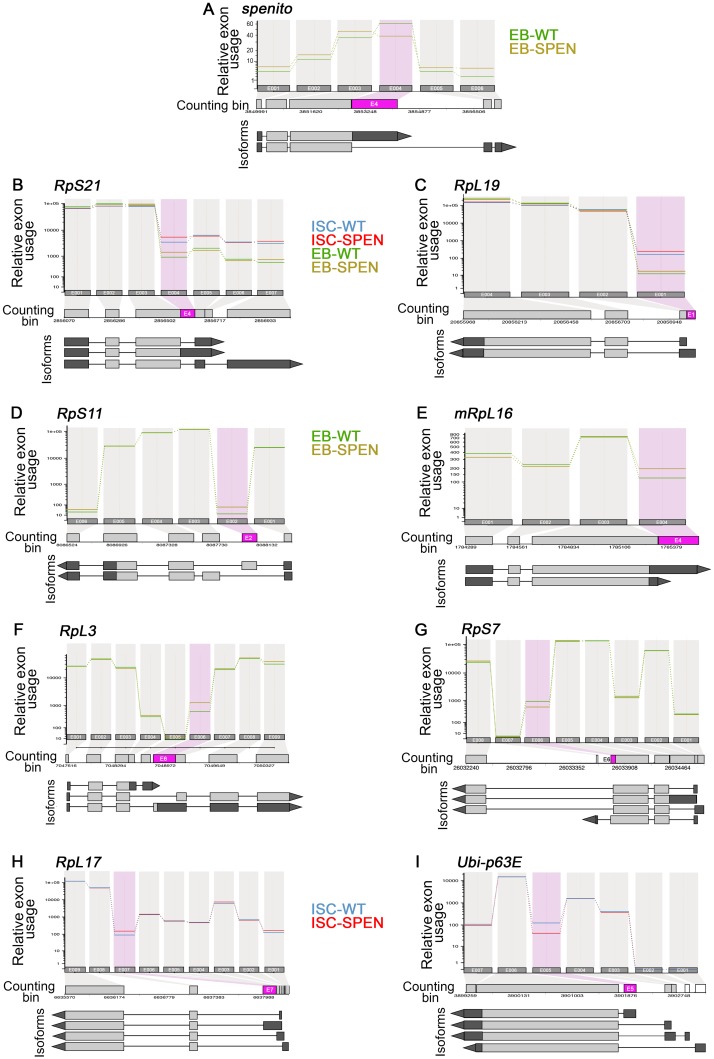
Examples of types of altered exons upon *spen* knockdown. Visualization of the relative exon usage, in a subset of example transcripts (DEXseq plot): (**A**) The relative exon usage of *spenito* was affected by *spen*^*RNAi*^ in EBs **(B)** Ribosomal protein encoding transcript RpS21 and **(C)** RpL19 had exon usage alteration upon spen^RNAi^ in both ISCs and EBs. **(D)** RpS11, **(E)** mRpL16, **(F)** RpL3 and **(G)** RpS7 showed differential exon usage in *spen* knock-down EBs. Additional examples of splicing could be found upon *spen*^*RNAi*^ expression in ISCs: within **(H)** RpL17 and **(I)** Ubi-p63E transcripts. On top, each line corresponds the relative exon usage of a single “counting bin” generated as defined in the Materiels & Methods. The relative exon usage is shown on top for each sample groups independently: ISC-WT (BLUE), ISC-SPEN (RED), EB-WT (GREEN), EB-SPEN (YELLOW). Significant differentially used exons (FDR of 0.05) are represented in PINK and non-expressed exons in both samples are represented in WHITE. Below, are shown the different annotated isoforms from each transcript, with the coding sequence (CDS) in LIGHT GRAY and the untranslated regions (UTRs) in DARK GRAY.

While the shorter SPEN-family members (Spenito in flies; RBM15, RBM15B in mouse) impact splicing regulation [33, 35, 37–39, 41], the larger SPEN-family proteins (*SPEN/SHARP* in mouse), to date, have primarily been implicated in transcriptional regulation [28, 31, 34, 76]. Nevertheless, mammalian SPEN was found as an interactor of the spliceosome, raising the possibility that SPEN also impacts splicing [29]. We therefore assessed exon usage in wild-type and *spen*^*RNAi*^ ISCs and EBs, and identified 141 transcripts that had one or more exon(s) with significantly different usage in ISCs and 193 in EBs (Fig 5K–5M, S5 and S6 Tables). We also found that the usage of 47 exons located in 37 transcripts, was similarly affected in the two cell types (Fig 5K, S5 and S6 Tables). Of note, the alterations in exon usage that we detected upon *spen* knockdown are comparable to the number of transcripts with altered splicing events upon inactivation of the Spenito-associated m^6^A methylation complex. Upon the inactivation of *ime4*, encoding a methyltransferase component of this complex, 163 transcripts were affected. Inactivation of, *YT521*, encoding an m^6^A reader altered 103 transcripts [33, 41]. Interestingly, our data also revealed that in EBs expressing *spen*^*RNAi*^, *spenito* exon E4, located in its 3’UTR, was significantly less used than in WT (Fig 6A). This may reflect an increase in the long 3’UTR *spenito* isoform due to E4 exon skipping. These observations suggested that *spen* affects exon usages of many RNAs including that of its related gene, *spenito*.

We then wanted to understand whether the majority of Spen’s activity in controlling gene expression was mediated by alteration in exon usage. Of the 55 genes altered upon *spen* knockdown in ISCs, 7 (16.7%) were found to have altered expression levels and exon usage. Similarly, of the 440 genes differentially expressed between wild-type and *spen*^*RNAi*^ in EBs, 25 genes (5.7%) were both differentially expressed and had altered exon usage (Fig 5L and 5M and S7 Table). This suggests that in absence of *spen* function, most of the changes in differential gene expression (83.3% in ISC and 94.3% in EBs) are not due to a difference in exon usage. Thus, we conclude that Spen controls transcript abundance and exon usage in ISCs as well as in EBs. In addition, Spen has common effects on transcripts independently of the cell type, which may reflect more general Spen target genes. Finally, Spen also has cell-specific effects on RNAs in ISCs and EBs.

### *spen* regulates genes involved in similar biological processes

In order to assess the function of the spen-dependent genes, we performed a GO term enrichment analysis on the differentially expressed (DE) genes and genes with alternative exon usage (AEU) in ISCs and in EBs respectively, upon *spen* inactivation (S3 Table). In ISCs, our analysis revealed 3 over-represented GO term categories associated with the symporter activity. Of note, these included Ca^2+^ transport regulators (*cac* [DOWN in ISCs], CG5348 [UP in ISCs], *pain* [down in EBs]) and regulators of Glutamate transport (*dmGLUT* [UP in ISCs and EBs], CG9864 [down in EBs], CG15096 [UP in EBs]) and the more general amino acid transporter *NAAT1* [UP in EBs]. Given the recent findings of regulation of ISC proliferation through L-Glutamate and Ca^2+^ influx [5, 77, 78], we hypothesize that the disruption of their expression upon *spen* inactivation may contribute to the defects in *spen* mutants of ISC numbers and proliferation. Furthermore, *cac* affects lysosomal transport [79], raising the possibility that its alteration could change the dynamics of Dl protein in *spen* mutant ISCs.

In EBs, the GO term enrichment analysis of the *spen*-dependent differentially expressed genes showed 10 over-represented GO term categories associated with enzymatic activity (S3 Table). It also revealed an over-representation of the GO term category “maintenance of imaginal disc-derived wing hair orientation” (S3 Table), which was supported by the upregulated genes *cora* (encoding an actin binding protein) and *Gli* (encoding a transmembrane protein known to localized at tricellular junctions in the intestine [80]). This function is affected by *spen* during *Drosophila* development, where it is required for the formation and the correct positioning of vein and bristle cells, as well as the maintenance of planar polarity in the wing imaginal discs [81]. Interestingly, *spen* knockdown led to alteration of transcript levels and exon usage in numerous ribosome associated genes. 4 over-represented GO term categories associated with cytoplasmic ribosomal processes were found of genes with alternative exon usage in EB upon *spen* knockdown. Indeed, upon *spen* knockdown in EBs, 13 ribosomal protein-coding genes had changes in their exon usage (Fig 6B–6I, S5 Table). In addition, 19 ribosomal protein-coding genes had differential gene expression and were found downregulated in EBs upon *spen*^*RNAi*^ (S4 Table). Among them, *RpS21* and *RpL19* were downregulated and had an alternative exon usage dependent of *spen* (S3 and S4 Tables). In ISCs, *spen* knockdown affected the exon usage of 8 ribosomal protein coding transcripts, including the *RpS21* and *RpL19* transcripts (S4 Table). Therefore, *spen* regulates a variety of genes encoding ribosomal proteins.

While not found as an enriched GO term, we also noticed that numerous *spen*-dependent genes in ISCs and EBs were related to chitin binding and chitin metabolism. In insects, chitin is a major component of the cuticle or exoskeleton and forms a barrier with the external environment. In *Drosophila*, the intestine is protected by both type I and type II peritrophic matrices with type I being secreted throughout the midgut and type II forming a sleeve-like membrane that is produced by the proventriculous. In *spen* knocked-down ISCs, the chitin binding proteins coding genes *CG14989*, *CG33258*, *Cpr51A*, and the Chitinase 2 coding gene *(Cht2*), were found downregulated, whereas the gene *yellow*, associated with cuticle pigmentation, was upregulated (S4 Table). In absence of *spen* function in EBs, *CG33258*, *Cpr51A* genes were downregulated as well, whereas the gene *ebony*, also associated with cuticle pigmentation, was upregulated (S4 Table). This may explain defects of *spen* mutants in cuticle integrity noted by others leading to defects in the formation of the sclerites (a cuticular structure), and in the trachea [43, 82, 83].

It is also of note that the expression of several genes related to immune response was altered upon *spen* inactivation. The Imd pathway is activated in the adult gut in response to bacterial infection [84] and inactivation of Imd pathway can stimulate ISC proliferation [7, 85, 86]. In *spen* knockdown ISCs, the immune response genes *AttC* and *pirk* were upregulated, as well as *Tg*, a transglutaminase that acts to inhibit the Imd component Relish [87]. Interestingly, the Pvr ligand Pvf1 was found to be upregulated in *spen* knockdown EBs. The PDGF and VEGF (Pvr) pathway regulates immune signaling through the IMD pathway [88]. Previous work has established that the overexpression of the secreted Pvf1 ligand in ISCs and EBs activates Pvr in the ISC and drives increased proliferation and expansion of the ISC population [89]. Therefore, the upregulation of Pvf1 in *spen* mutant EBs may promote increased ISC proliferation and accumulation. In addition, other immune response genes were also upregulated, including bactericidal factor-encoding genes *LysB*, *LysE* and *LysD* from the lysozyme locus, the gene coding the peptidoglycan recognition protein PGRP-SC2, and also additional downstream effectors of the Imd pathway: *relish/NF-kB* and *pirk* (S4 Table). Moreover, alternative exon choice of *PGRP-LB* was detected in *spen* knockdown EBs (S5 Table). Thus, the Imd pathway may be affected by the inactivation of *spen* function in the intestine. This could explain some phenotypes of *spen* mutant larvae that have defects in the immune response [50, 90]. Altogether, these data suggest that Spen regulates genes associated with similar functions that may impact on intestinal homeostasis and physiology.

## Discussion

**Fig 7:**
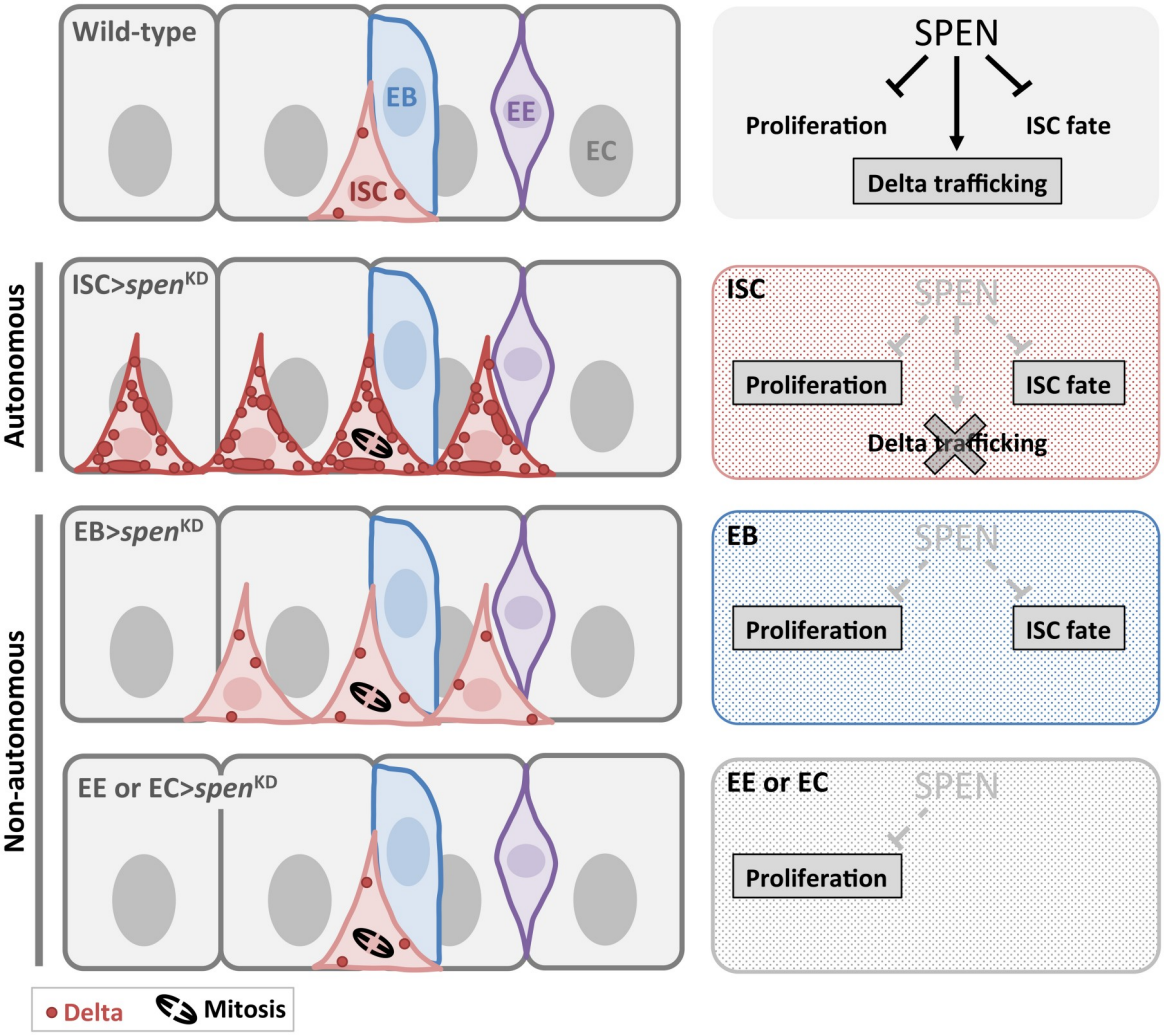
Model. Autonomous and non-autonomous roles of *spen* in the control of intestinal stem cells. In the intestinal stem cell (ISC), *spen* acts autonomously to limit their proliferation and stem cell self-renewal. Its inhibition leads to an accumulation of the Delta protein at the membrane. This is likely due to an effect on Dl trafficking, which may be direct or indirect. *spen* also acts in a non-autonomous manner in the Enteroblast (EB) to limit ISC numbers. Furthermore, *spen* inactivation in EBs, Enteroendocrine cells (EE) and Enterocytes (EC) leads to enhanced ISC proliferation. Further studies will be required to better understand Spen’s molecular functions.

The mechanisms by which ISCs undergo asymmetric and symmetric divisions are still not completely understood. Here, in a genetic screen, we have identified *spen* as an essential regulator of adult stem cells in *Drosophila*. Our data indicate that in the absence of *spen* activity, stem cells have aberrant high levels of Delta (Dl) protein and fail to properly commit into EB daughter cells resulting in large increase in numbers of ISC-like cells and that this activity is cell autonomous roles in the ISC. Furthermore, *spen* acts in EBs to limit ISC numbers, and in EBs, EEs and ECs cells to suppress ISC proliferation. Therefore, in *spen* mutant tissue, stem cell autonomous and non-autonomous mechanisms that act to drive ISC proliferation, increased ISC numbers, and aberrant accumulation of Dl protein in ISCs (Fig 7). While *spen* mutants are dependent on Akt and InR for their growth, they are not as sensitive to a reduction in EGFR activity as wild-type control clones are. Our findings argue that Spen does not act as a general regulator of Notch signaling in all tissues, and is upstream of, or parallel to, Notch pathway activation to promote intestinal stem cell commitment. Through a transcriptomic analysis, we have identified genes controlled by *spen* in ISCs and EBs, both at the transcript and exon level, providing candidate ISC regulators downstream of *spen*. Critically, this work shows that the RNA binding protein Spen is an important regulator of asymmetric fate outcomes of ISC division and its proliferation.

It is interesting that *spen* has both stem cell-autonomous and non-autonomous activities to regulate stem cell numbers and proliferation (Fig 7). The knockdown of *spen* in ISCs and EBs can expand stem cell numbers, whereas the knockdown in EEs and ECs led to a dramatic increase in the number of dividing ISCs, but did not change the density of ISCs. We believe, therefore, that the growth of *spen* mutant clones is influenced by both cell autonomous and non-autonomous processes: *spen* inactivation in the stem cell leads to more ISC symmetric self-renewing divisions, however, EB, EE and EC cells are still produced. The mutant EB, EE, and EC cells can further drive proliferation of the extra ISCs through non-autonomous regulation. It is possible that the non-autonomous regulation of ISC proliferation detected upon downregulation of *spen* in EEs and ECs could be due to stress induced in the tissue owing to disruption of numerous *spen* targets genes in these cells. Importantly, our findings highlight the complex process of stem cell deregulation arising in tumor contexts whereby inactivation of a tumor suppressor genes may have numerous functions in different cells within a tumor that collaborate to drive tumorigenesis.

One intriguing effect of *spen* inactivation is the high levels of accumulation of the Notch ligand Dl at the plasma membrane and in intracellular vesicles. Our RNAseq data revealed that Dl mRNA levels or exon usage are not affected, suggesting a regulation at the protein level, perhaps during its endocytic trafficking steps. This phenotype of Dl protein accumulation is reminiscent of those occurring upon Dl trafficking perturbation, such as in *neuralized* mutants [58, 91, 92]. A previous study has shown genetic interaction of *spen* and regulators of trafficking of Notch ligands [93]. Indeed, in the developing *Drosophila* eye disc, *spen* genetically interacts with the endocytic adaptor *Epsin*/*liquid facets* (*lqf*), which promotes Dl trafficking and internalization facilitating Dl activation [94, 95]. Spen, however, is likely not a general regulator of protein trafficking as the localization of the membrane protein Sanpodo occurred normally in *spen* mutant clones in the intestine. Therefore, Spen likely regulates Dl trafficking in a tissue-specific manner and indirectly through transcriptional or post-transcriptional control of a downstream gene.

In addition, our data indicated that the knockdown of Akt and InR activity could reduce the number of Dl+ ISCs produced in the *spen* mutant background. The InR pathway has previously been implicated in regulating symmetric cell divisions of the ISC during adaptive growth [59]. Thus, it appears as if Akt and InR are also facilitating symmetric cell division in the *spen* mutant context. Our data also show that *spen* mutant stem cells are insensitive to a reduction of EGFR signaling upon overexpression of EGFR-DN, suggesting that *spen* and EGFR pathway have opposing roles in regulating ISC proliferation. While a decrease in EGFR signaling reduces ISC proliferation, *spen* inactivation, in contrast, leads to an increase in proliferation. *spen* has been shown to genetically interact with the EGFR signaling pathway during *Drosophila* embryogenesis and during eye development [44, 46, 48, 51, 96, 97]. Nevertheless, in these contexts, *spen* potentiates EGFR signaling. Thus, *spen* may have a tissue specific effect regarding EGFR pathway regulation. The precise link between *spen* and EGFR signaling regulation is nevertheless still unclear. Indeed, while a constitutive active form of Ras can rescue the lethality caused by a *spen* dominant negative form in embryo [48], *spen* seems to act downstream or in parallel of Ras activation during photoreceptor specification [44, 46, 48, 49, 96, 97]. Additionally, *spen* loss of function in cone cells during pupal eye development affects the EGF receptor ligand Spitz level, which suggest a role at the level of the EGF receptor activation [51]. Further studies will be required to better understand the link between *spen* and the EGFR signaling pathway in the regulation of intestinal stem cell regulation, as well as in other developmental contexts.

In addition to describing a new function of *spen* in adult stem cell regulation, our data raise a number of interesting questions about the function of *spen* and its downstream target genes in stem cell regulation. Importantly, we demonstrate that inactivation of a large SPEN family member also results in considerable alteration of exon usage, like the smaller family members (*spenito* in flies, *RBM15* and *RBM15b* in mouse). Thus, *spen* activity is important for transcriptional and post-transcriptional regulation in *Drosophila*, although we cannot exclude that some of these effects might be indirect. Interestingly, in absence of *spen* function, altered exon usage of *spenito* was detected in EBs, raising the possibility of feedback control between family members, reminiscent of the plant SPEN family member FPA [30].

Little is known about the impact on intestinal stem cell self-renewal of RNA binding proteins. A recent study found that inactivation of *Tis11*, encoding a regulator of RNA stability, led to larger clones than controls [98]. The *Tis11* phenotype differs markedly from that of *spen* as it appears not to affect cell fate decisions since ISCs do not accumulate as in *spen* mutant clones. Importantly, our study provides a data set that opens new perspectives for future investigation on the relationship between Spen and RNA regulation at transcriptional and post-transcription levels. Interestingly, our data also reveals that Spen co-regulates several genes encoding proteins that are involved in similar biological processes, such as cytoplasmic ribosomal proteins, immune response pathway and Imd pathway genes, and genes involved in Chitin metabolism. This may explain previously reported *spen* phenotypes in other tissues [43, 46, 48, 49, 56, 82, 90, 99].

Recent studies have highlighted important, essential functions of Spen family proteins from X-inactivation in mammals to sex determination and fat metabolism in flies [33, 35, 41, 47, 100]. This family of proteins is also frequently mutated in cancers, though mechanistically the role SPEN family proteins in cancers is not understood [101–106]. Our work demonstrates that *spen* inactivation causes the deregulation of numerous genes and results in alteration of the stem cell fate acquisition. In mammals, RBM15 has a critical function in promoting hematopoietic stem cell return to quiescence [52]. These findings raise the possibility that conserved functions of SPEN family proteins may help restrict stem cell activity required for cancer prevention.

## Materials and methods

### Fly stocks

Fly stocks used in this study were the following:

*FRT40A spen*^*9E34*^ [allele presents a nonsense mutation (C>T at genome position 2L:185541), which leads to a putative 630 aa truncated protein]; *FRT40A spen*^*9C95*^ [allele presents a nonsense substitution (C>T, 2L:190065), which leads to a putative 1961aa truncated protein]. The 9E34 and 9C95 mutations were mapped with Sanger and Illumina whole-genome sequencing, respectively. Of note, our fly stock containing the *spen*^*9E34*^ allele was lost from our collection in 2017, though this allele produced identical phenotypes to the *spen*^*9C95*^ and *spen*^*5*^ allele, which were primarily used in our study. From Bloomington Drosophila Stock Center (BDSC): *UAS-AKT*^*RNAi*^ (#BL33615); *UAS-InR-DN* (BL8253), *FRT40A spen*^*5*^ (#BL8734) [43]. From the Vienna Drosophila RNAi Center (VDRC): *UAS-spen*^*RNAi*^ *(#KK-108828)*. The lines *NRE-Venus* and *Su(H)GBE-LacZ* (*NRE-LacZ*) [57, 71], *UAS-Notch*^*cdc10*^ [referred as *UAS-N*^*act*.^; is a truncated, active version of intracellular Notch [107]. *UAS-EGFR-DN* (the 3^rd^ chromosome of BL5364 was used) The *spen* rescue experiment was performed using the pACMAN construct CH321-56B18 from [108], inserted on the 3 chromosomes attP-76A2. UAS-spen7RNAi, gift from I. Rebay Rab5::YFP, Y. Bellaiche, *pros*^*voila*^*-Gal4* [109]; p[Myo1AGal4]NP0001, gift of H. Jiang, *UAS-spen7*^*RNAi*^, K. Cadigan, *Tub-Gal4-GeneSwitch*, M. Rera.

For thermosensitive RNAi expression, the following lines were used:

*esgGAL4*, *tubGAL80ts*, *UAS-GFPnls (Chr2)* (*esg*^*ts*^; Jiang et al., 2009); *esg-GAL4*, *tub-GAL80*^*ts*^; *UAS-RFP* (made with line BL31417 from Bloomington stock center); *NRE-GAL4* [110]), *UAS-CD8-GFP*; *tubGAL80*^*ts*^ (*NRE*^ts^) and *esg-GAL4; NRE-GAL80*, *tub-GAL80*^*ts*^ *NRE-GAL80* (*esg-GAL4*,*UAS-YFP; NRE-GAL80*, *tub-GAL80*^*ts*^ [111] kindly received from B. Edgar).

MARCM clones were generated using the following stock:

*w P[hs-FLP] P[pTub-GAL4] P[UAS- nlsGFP]; FRT40A P[pTub-GAL80]* and *w P[hs-FLP] P[pTub-GAL4] P[UAS- nlsGFP];;FRT82B P[pTub-GAL80]*. MARCM^ts^ clones were generated using the following stock: *w P[hs-FLP] P[pTub-GAL4] P[UAS- nlsGFP]; FRT40A P[pTub-GAL80]; P[pTub-GAL80ts]*.

### Mosaic analysis with a repressible cell marker (MARCM) and RNAi experiments

Adult flies and crosses were kept at 25°C in rich yeasted medium tubes unless otherwise noted. MARCM clones were induced by a 35 min. heat shock at 36.5°C in 3 day old adults. Flies were then dissected either at 5 days, 6 days or 10 days AHS. For MARCM^ts^ experiments in Fig 3K–3P were done with the following modifications: Crosses and adults were kept at 18°C before and after heat shock. 10 days AHS, adults were shift at 29°C for 4 days and then dissected. RNAi thermosensible experiments were kept at 18°C and shifted at 29°C during indicating period before dissection. For GeneSwitch experiments (Fig 5G, S3 Fig), crosses and newly eclosed siblings were raised in a standard food. 3 days old adults were sorted and shifted on food supplemented with 50 μg/ml of RU486 in EtOH or EtOH alone, at 25°C during 2 days prior dissection.

### Immunofluorescence and confocal microscopy

Antibodies used in this study: anti-Delta ECD C594.9B [mouse, ascites, 1/2000, Developmental Studies Hybridoma Bank (DSHB)]; anti-PH3 (Rabbit, 1/2000; Upstate); anti- Pros (Mouse, 1/2000; Y. N. Jan, UCSF, CA, USA); anti-Pdm1 (Guinea pig, 1/1000; W. Chia, National University of Singapore); anti-Sanpodo (1/2000; preabsorbed; J. Skeath, Washington University, St Louis, MO, USA); anti-Notch ECD C458.2H (mouse, ascites, 1/100, DSHB); anti-βGal (Goat, 1/500; Biogenesis); anti-GFP (Chicken, 1/2000, Molecular Probes). The Delta (C594.9B) antibody developed by S. Artavanis-Tsakonas, and the Prospero (MR1A) antibody developed by C.Q. Doe were obtained from the Developmental Studies Hybridoma Bank, created by the NICHD of the NIH and maintained at The University of Iowa, Department of Biology, Iowa City, IA 52242. The fixation protocol previously described in [25] was used in this study. A methanol fixation protocol described in [112], was used for anti-Notch immunofluorescence in Fig 1K and 1L.

### Quantification and statistical analysis

All experiments were performed on female flies. For clonal analyses of this study, posterior midguts were analyzed and “stem cell clones” (i.e. clones deriving from ISCs and containing two or more cells) were scored. In Fig 3E and 3F, single cell clones were also included in the analysis since stem cell clones differentiate under expression of active Notch. In Fig 2, Delta+ cell density referred to the number of Delta+ cell per pictures area (1000μm^2^) and the number of PH3+ cells was quantified in the posterior midgut. Statistical analyses were performed with GraphPad Prism version 5 for Mac, GraphPad Software (La Jolla California USA, www.graphpad.com) using the nonparametric Mann-Whitney two-way ANOVA, when not specified. In Fig 1Q, a Fisher’s test was performed. For Fig 4, data from 2–4 experiments were combined, therefore some of the data points from wild-type and *spen* mutant clones are the same in graphs of Fig 4E–4J and 4L–4N. We note that in the experiments at 10d in Fig 4, while *spen* clones had a greater mean size than wild-type, this difference was not significant in a Mann-Whitney test which measures median values, in contrast to 5d clones in Fig 1 where this difference was significant. This may be due to the fact that at 10d, cells within clones are undergoing turnover. Nevertheless, the number of clones >29 cells, was significantly different in *spen* compared to controls at 10d (p>0.05, Mann-Whitney).

### Intestinal cell FACS sorting

The following genotype was used: *esg-GAL4*, *tub-GAL80ts*/ (*UAS-spen*^*KK*^ *RNAi* or *+); UAS-RFP*, *NRE-Venus* expression was induced by a 2 day temperature shift from 18°C to 29°C. Entire intestines were dissected and collected in cold PBS. Dissociation was performed as described in [113] using PBS-Elastase (1mg/ml) solution and mechanical pipetting. Dissociated cells were resuspended in PBS, 1% BSA and FACS (FACS Aria BD Biosciences) using the following parameters: 20psi, 100μm nozzle. Diploid RFP positive fraction were separated from the diploid RFP, GFP double positive fraction. Cells were directly collected in TRIzol (Life Technologies).

### RNA-seq

RNAs was extracted by performing two series of Phenol-Chloroform extractions at 4°C, followed by DNase treatment according to the manufacturer’s instructions. ExpressArt Pico RNA Carrier compounds for very small samples was used to precipitate the RNA. Around 100ng total RNA was collected from 100,000 sorted cells. Total RNA quality and quantification were assessed on an Agilent Bioanalyzer using the Agilent RNA 6000 nano kit. An Illumina TruSeq Stranded RNA (LS) protocol was used to build the libraries, from polyA RNAs enriched by Oligo-dT bead polyA tail capture. Due to a failure of one of the EB-WT samples to cluster with the other two samples during the Principal component analysis; only two biological replicates were used for the EB-WT whereas three biological replicates for the others conditions. Sequencing was performed on the Illumina HiSeq 2500.

### RNA-seq analysis

For the following analyses, default parameters were used if not indicated, associated with a paired-end and first strand library type modes. *Alignment—*Sequencing resulted in about 50 million 100bp paired-reads from each library, which were then aligned against the *Drosophila melanogaster* r5.54 genome reference using TopHat (version 2.0.6) with the following parameters (-N 3—read-edit-dist 3 -p 2 -g 1) [114]. Unmapped reads were discarded. *Counting reads—*Mapped reads were counted on gene using SummarizOverlaps method from the GenomicAlignments package (version 1.8.0) [115] with a “Union” mode, based on the ENSEMBL GENES 75, BDGP5 genome reference.

Principal component:

Principal component analysis was performed using the PCAplot function from DEseq2 R package on transformed normalized read counts (rlog function) of all samples. This analysis considers the 300 top-ranked genes (ntop = 300) with the highest row variance across all samples. PCA showed that our samples were distinguishable by the first and second principal component (PC1, 44% and PC2, 19% of the total variation). Interestingly, when doing the PCA analysis by selecting the 500 top-ranked genes based on their row variance, ISC and EB populations were clearly discriminated by the first principal component (PC1, 39% of the total variation), but not the difference between WT and SPEN samples by the second principal component (PC2, 19% of the total variation).

Differential expression analysis:

Differential gene expression was analyzed using DEseq2 (version 1.12.2) [116]. The following parameters: DESeq(minReplicatesForReplace = Inf) and results(cooksCutoff = FALSE) were used in order to disable the Cook’s filtering and flagging. Genes were defined as differentially expressed according to their padj<0.5.

Differential exon usage:

Differential exon usage was analyzed using DEXseq (version 1.18.4) with default parameters [75]. With this method developed by Anders et al 2012, we assessed the changes in relative exon usage independently of the overall differential gene expression. During the read mapping process, in the case where exons do not share the same boundary in all transcripts, DEXseq splits the exon in two or more parts to create “counting bin”. Thus, this term refers to exons or parts of exons generated from this manipulation. Exons with an adjusted p values (padj) less than 0.5 were considered as significantly differentially used. In order to visualized the relative exon usage without the impact of the change in gene expression level, we used the function “plotDEXSeq” with the option “splicing = TRUE” provided by DEXseq, which removes the effects due to changes in gene expression from the plots. Venn diagrams were generated using Venny 2.1.0 tool (Oliveros, J.C. 2007–2015).

The RNAseq data produced from this publication have been deposited to the NCBI GEO and are available under accession number GSE84367.

### GO enrichment analysis

The GO term gene annotation was done with the PANTHER Classification system [117] developed by the Gene Ontology Consortium [118].

GO term enrichment test—The R package GOseq version 1.28.0 (released 3.5, 2016-05-30) [119] was used to perform a GO term enrichment test. Each set of differentially expressed genes were compared with the list of 11,103 expressed genes in the ISC and EB as a background. This list of background genes was obtained by considering as an expressed gene, a gene with non-zero FPKM in at least 1 sample in either WT-EB or WT-ISC conditions. Thus, all unexpressed genes were removed from the list of total number of gene in the *Drosophila melanogaster* genome (r5.54). Only pval<0.05 are shown.

### RT-qPCR validation

To further validate the relative change in gene expression of some of these genes, we ubiquitously expressed *spen*^*RNAi*^ during 2 days using the *tubulin-GeneSwitch* driver (*tubGS*, [120]), and tested their change in relative expression by RT-qPCR from whole midguts. In this condition, ISC-like cells expressing *spen*^*RNAi*^ showed an enrichment of Delta protein at the membrane, but no major accumulation of ISC-like cells (S3A–S3D Fig). Thus, a change in relative expression would reflect an intrinsic change in gene expression *per se* due to *spen* knockdown, and not a simply increase in ISCs per gut. As a reference gene, we used *rp49* gene, which showed a constant expression over the different conditions (S3E Fig).

RNA from 25–30 whole female midguts was extracted using TRIzol (Invitrogen) followed by two series of Phenol-Chloroform extractions at 4°C, and a DNase treatment according to the manufacturer’s instructions. cDNA was synthesized using the iScript cDNA synthesis kit (BioRad). RT-qPCR was performed using SYBR Green (SYBRPOWER) on a ViiA 7 real time PCR system (Thermofisher). RT-qPCR was performed in triplicate on each of three independent biological replicates. rp49 was used as a normalization control. The relative expression was calculated based on the Livak Method. The FoldChange was calculated between the SPEN.RU and WT.RU samples from the same experiment. Primers used for the RT-qPCR are the following: rp49-F1 CCGCTTCAAGGGACAGTATCTG; rp49-R1 ATCTCGCCGCAGTAAACGC; CG5348-F TTCATCGCCTTGTTCGTGG; CG5348-R GGGATACGGCCTTCAGAGC; unc-104-F GTTCACTCGCATACAGGATACTG; unc-104-R CTGACTCGCTCGCAATAGATTT; dmGLUT-F GGATGACGATTCTGAGGCGAT; dmGLUT-R GAATGAGGACAGGATCAAACCTT; pirk-F ATGGGCGTTCGTGTGATAGAA; pirk-R TTACCCTGCTCGTGCTCTTTC; tej-F GACGCCGAATCCTTTCTCCG; tej-R GCAGTTATCCATGCCATTTGTCC; Tg-F TCAGCAGTATTGGGCGTTTTG; Tg-R GCATAGAAGTGACTCGTATGGTG; neb-F ACAGGAATGCGAACTCTACTCC; neb-R CTGAGGCAGGACTCCATCAGA; CG9505-F ACAGAGTTGTCCGTACCTCG; CG9505-R CGTAGGCATAGAAATCCTCGC; CG11190-F AGCTAGTGGTTCGTCCATTGT; CG11190-R AGATTATCCTTTTCGCCGTAGTG; Cpr51A-F TCAACGATGGACAAATCTCACG; Cpr51A-R TTATCGGCGATGTACTCAACAC; CG33774-F GTTCTGGGCGTATTTCTGTTCC; CG33774-R CACTGGGCTTTCCGGTATTGG; CG14989-F ATGGGAGAGCGTGTTGAAAAG; CG14989-R TTGTGACCAATGGTATGACGAG; Ir85a-F CCAGTGGCTAAAACACATTCTGC; Ir85a-R AGGATCTGACTCATCTTGACCA; CG11241-F: CAAGCGAGTGTTAGCTCAGAC; CG11241-R: TGGATCACAAGTGGCTTTTTGAA; Aats-ser-F: ACAGTCCAGGCTCAAGGAGTT; Aats-ser-R: CAGTGTAGTTACCCAAGTGATCC.

## Supporting information

S1 Table Differentially expressed genes: ISC versus EB. Genes that were differentially expressed between ISC and EB cells.
(XLSX)

S2 Table Raw data DEseq2 analysis. Raw data of analysis of differential gene expression using DEseq2 comparing wild-type ISCs vs EBs, *spen* knocked-down ISC versus wild-type ISC, and *spen* knocked-down EB versus wild-type EB.
(XLSX)

S3 Table Go term enrichment. Go term analysis of differentially expressed genes in wild-type ISC-enriched genes; of differentially expressed genes in *spen* knocked-down ISC versus wild-type ISC; of differentially expressed genes in *spen* knocked-down EB versus wild-type EB; and of altered exon usage in *spen* knocked-down EB versus wild-type EB.
(XLSX)

S4 Table Differentially expressed genes: *spen*-dependent genes. Genes with significant changes (FDR of 0.05) upon *spen* knockdown with indicated Log2FC and associated biological term. Differences between *spen* knocked-down ISC versus wild-type ISC, and *spen* knocked-down EB versus wild-type EB are shown.
(XLSX)

S5 Table *spen*-dependent altered exon usage. Genes resulting from DEXseq analysis that demonstrated significant altered exon use upon knock down of *spen* in ISCs and EBs.
(XLSX)

S6 Table Raw data DEXseq analysis. Raw data of analysis of altered exon usage using DEXseq comparing wild-type ISCs vs EBs, *spen* knocked-down ISC versus wild-type ISC, and *spen* knocked-down EB versus wild-type EB.
(XLSX)

S7 Table Genes with differential gene expression and altered exon usage. Genes that were both differentially expressed and had altered exon usage in ISCs and EBs, wild-type versus *spen* knockdown.
(XLSX)

S1 Fig *pros*^*voila*^*-GAL4* and *myo1A-GAL4* drivers showed weak expression in some Dl+ cells. Related to Fig 2 *wild-type UAS-GFP* controls (A- B’”, F -H”‘) and *UAS-spen*^*RNAi*^ (C- E’”, I-K”‘) expressed in enteroendocrine cells **(A-E”‘)** or in Enterocytes **(F-K”‘)** using *pros*^*voila*^*-GAL4* or *myo1A-GAL4*, respectively, for 10 days at 29°C. Single focal plane images shown. Arrowheads show Delta+ ISC cells with weak GFP expression. GFP in GREEN marked cell type expression, ISC-like cells (Delta+, RED), Enteroendocrine cells (Pros+, RED nuclear), mitotic cells (PH3+, Gray), DNA (DAPI, BLUE). Scale bar: 10μm.
(TIF)

S2 Fig Genetic interaction between *spen* and *Insulin receptor (InR)*. **(A-B).** Related to Fig 4 **(A)** large *spen*^*5*^ clones, were reduced in size upon expression of an *InR*^*D*N^ construct **(B)** *spen*^*5*^*; InR*^*DN*^ clones, 10d after heat shock (AHS). Some cells showed Delta accumulation at the membrane (Delta+, RED; GFP, GREEN; DAPI, BLUE). **(C)** Quantification of cells per clone, **(D)** Dl+ cells per clone, and **(E)** Dl cell proportion per clone in A-B. (**F**) Percent of Dl+ cells per clone. p<0.01, **. p<0.001, ***. p<0.0001, ****. Mann-Whitney Two-Way ANOVA test. Error bars represent the Standard Error of the Mean (sem). Scale bar: 20μm.
(TIF)

S3 Fig Whole gut expression of spe*n*^RNAi^. (**A-D**) Whole intestines used for RT-qPCR validation that ubiquitously express *spen*^*RNAi*^ during 2 days using the *tubulin-GeneSwitch* driver (*tub*^*GS*^) or control gut (*tub*^*GS*^*/+*), with Ethanol (RtOH) or RU induction. Scale bar: 20μm. (**E**) Relative expression (Mean of Ct values) of *rp49* gene by RT-qPCR. *rp49* gene showed a constant expression over the different conditions.
(TIF)
