# Correction: Spen limits intestinal stem cell self-renewal

**DOI:** 10.1371/journal.pgen.1010679

**Published:** 2023-03-03

**Authors:** The PLOS Genetics Staff

The Funding Disclosure section was missing the following funding information:

Lara Al-Zou’abi was supported by the Institut Curie 3-i PhD Program under the Marie Skodowska-Curie Grant Agreement No 666003.
