# Supplementary figures and images for: Spen limits intestinal stem cell self-renewal
Source: PLoS Genet. 2018 Nov 19;14(11):e1007773. doi: 10.1371/journal.pgen.1007773 (PMC6277126; doi:10.1371/journal.pgen.1007773)

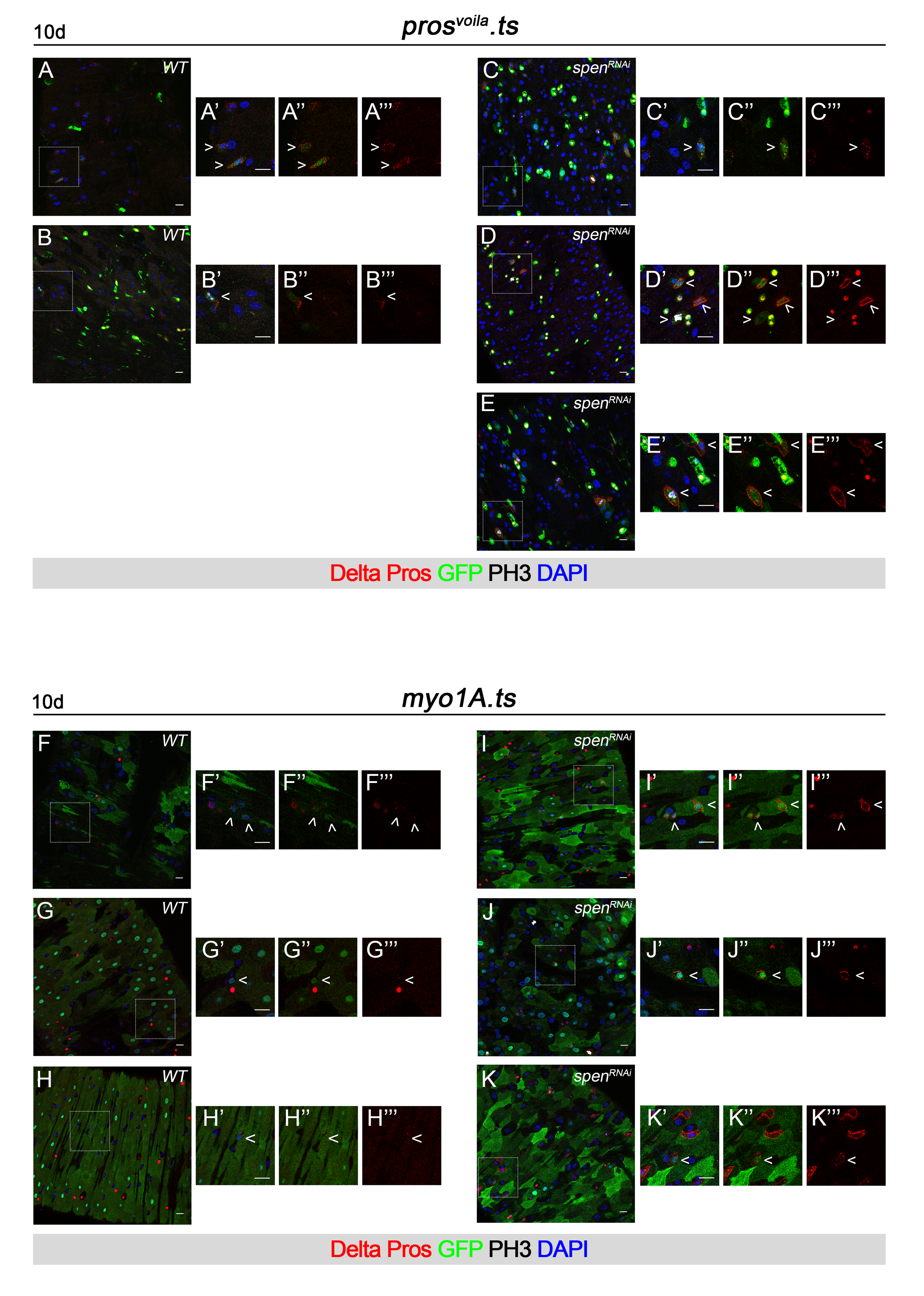

Supplement: S1 Fig — Related to Fig 2 wild-type UAS-GFP controls (A- B’”, F -H”‘) and UAS-spenRNAi (C- E’”, I-K”‘) expressed in enteroendocrine cells (A-E”‘) or in Enterocytes (F-K”‘) using prosvoila-GAL4 or myo1A-GAL4, respectively, for 10 days at 29°C. Single focal plane images shown. Arrowheads show Delta+ ISC cells with weak GFP expression. GFP in GREEN marked cell type expression, ISC-like cells (Delta+, RED), Enteroendocrine cells (Pros+, RED nuclear), mitotic cells (PH3+, Gray), DNA (DAPI, BLUE). Scale bar: 10μm. (TIF) [file pgen.1007773.s008.tif]

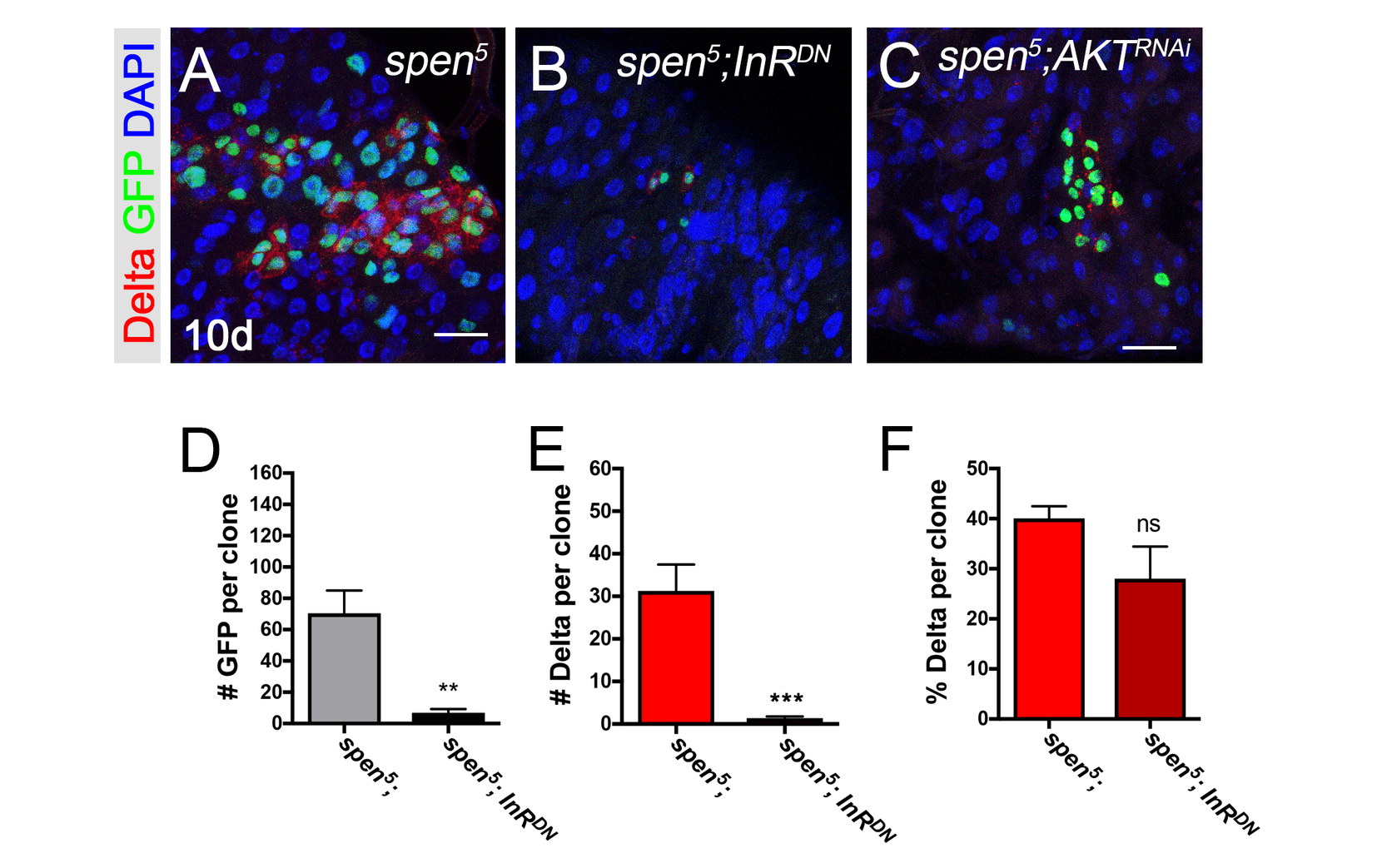

Supplement: S2 Fig — (A-B). Related to Fig 4 (A) large spen5 clones, were reduced in size upon expression of an InRDN construct (B) spen5; InRDN clones, 10d after heat shock (AHS). Some cells showed Delta accumulation at the membrane (Delta+, RED; GFP, GREEN; DAPI, BLUE). (C) Quantification of cells per clone, (D) Dl+ cells per clone, and (E) Dl cell proportion per clone in A-B. (F) Percent of Dl+ cells per clone. p<0.01, **. p<0.001, ***. p<0.0001, ****. Mann-Whitney Two-Way ANOVA test. Error bars represent the Standard Error of the Mean (sem). Scale bar: 20μm. (TIF) [file pgen.1007773.s009.tif]

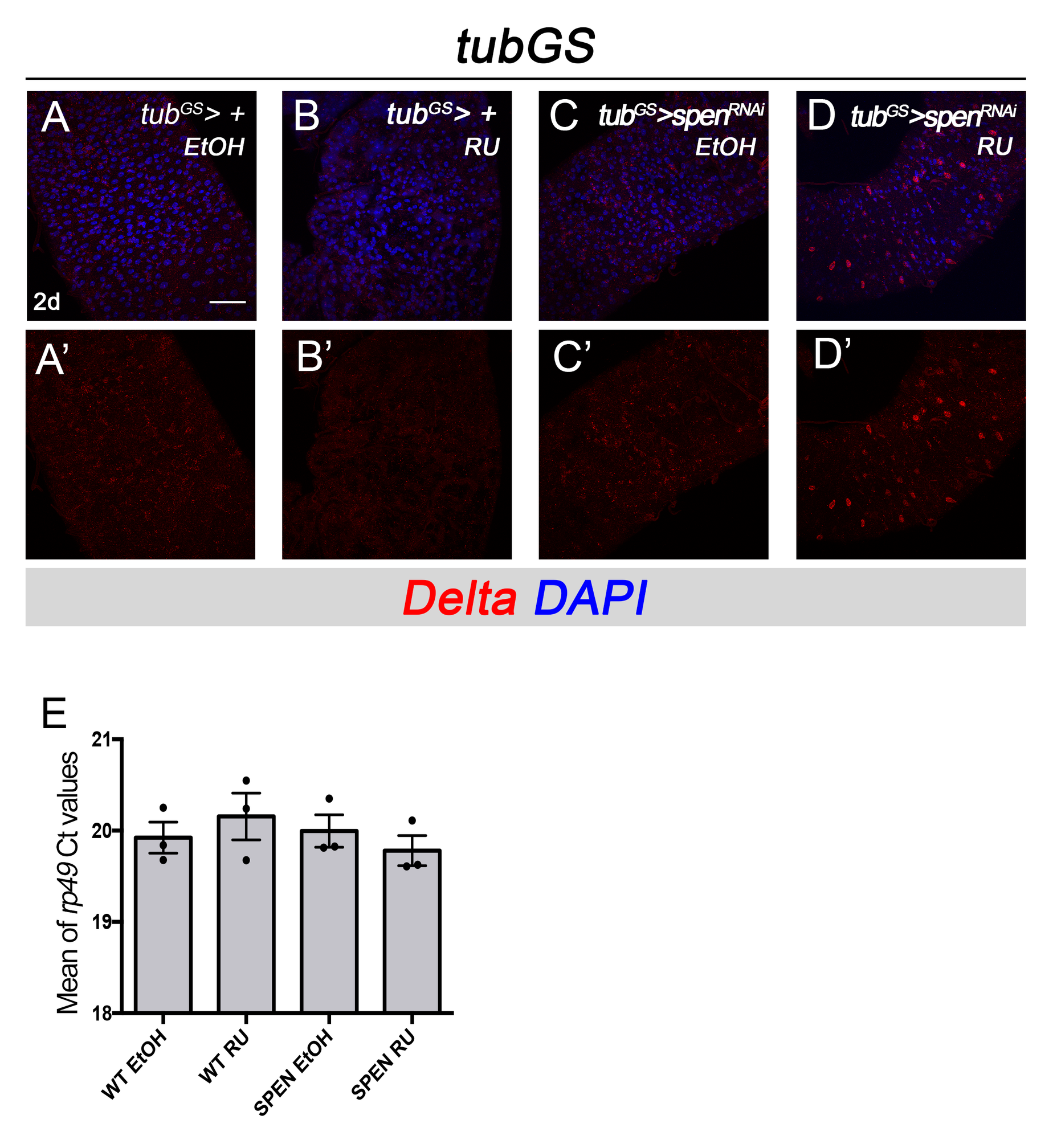

Supplement: S3 Fig — (A-D) Whole intestines used for RT-qPCR validation that ubiquitously express spenRNAi during 2 days using the tubulin-GeneSwitch driver (tubGS) or control gut (tubGS/+), with Ethanol (RtOH) or RU induction. Scale bar: 20μm. (E) Relative expression (Mean of Ct values) of rp49 gene by RT-qPCR. rp49 gene showed a constant expression over the different conditions. (TIF) [file pgen.1007773.s010.tif]
